# Multimodal Operando Characterization of Cation Effects at the Iridium Oxide–Electrolyte Interface for Alkaline Water Oxidation

**DOI:** 10.1021/acsami.5c22249

**Published:** 2026-03-04

**Authors:** Yemin Tao, Tomohiko Utsunomiya, Haiting Yu, Seung-Jae Shin, Caiwu Liang, Yifeng Wang, Aron Walsh, James R. Durrant, Mary P. Ryan, Yu Katayama, Aliaksandr S. Bandarenka, Reshma R. Rao

**Affiliations:** † Department of Materials, 4615 Imperial College London, Exhibition Road, SW7 2AZ London, U.K.; ‡ Department of Energy and Environmental Materials, SANKEN, The University of Osaka, Mihogaoka 8-1, Osaka, 567-0047 Ibaraki Japan; § Department of Physics, 13013 Technical University of Munich, James-Franck-Straße 1, 85748 Garching, München, Germany; ∥ School of Energy and Chemical Engineering, 131639 Ulsan National Institute of Science and Technology (UNIST), Ulsan 44919, Republic of Korea; ⊥ Department of Chemistry, Imperial College London, White City, W12 0BZ London, U.K.; # Grantham Institute—Climate Change and the Environment, Imperial College London, Exhibition Road, SW7 2AZ London, U.K.

**Keywords:** electrocatalysis, water splitting, electrode−electrolyte interface, oxygen evolution reaction, operando spectroscopy

## Abstract

Understanding the electrode/electrolyte interface is essential for tuning electrocatalyst activity. Here, we combine operando optical spectroscopy, laser-induced current transient (LICT) measurements, and surface-enhanced infrared absorption spectroscopy (SEIRAS) to investigate the origin of cation-dependent oxygen evolution reaction (OER) activity on electrodeposited iridium oxide in 0.1 M MOH (M = TMA^+^, K^+^, Na^+^, and Li^+^). We find that OER activity increases with increasing cation size (TMAOH > KOH > NaOH > LiOH). Operando optical spectroscopy reveals that the energetics of the redox transitions and the population of the redox-active species are independent of the electrolyte. Instead, the intrinsic turnover frequency varies strongly with the nature of the cation. LICT, SEIRAS, and quantum mechanics/molecular mechanics (QM/MM) simulations suggest that the interfacial solvent structure is the origin of this difference. With increasing cation size, the fraction of isolated water molecules and cation-coordinated water molecules increases, producing a more disordered interfacial environment. LICT measurements confirm that the potential of maximum entropy shifts closer to the water oxidation potential in the presence of larger cations in the electrolyte. We propose that a more disordered interface results in more isolated and reactive OH^–^ ions and faster reorganization of the interfacial solvent structure during the rate-determining O–O bond formation step, thereby accelerating the OER kinetics. Through our work, using multimodal operando spectroscopy and molecular simulations, we highlight how interfacial solvent structure, controlled by electrolyte cations, governs reactivity at complex electrochemical interfaces.

## Graphical abstract

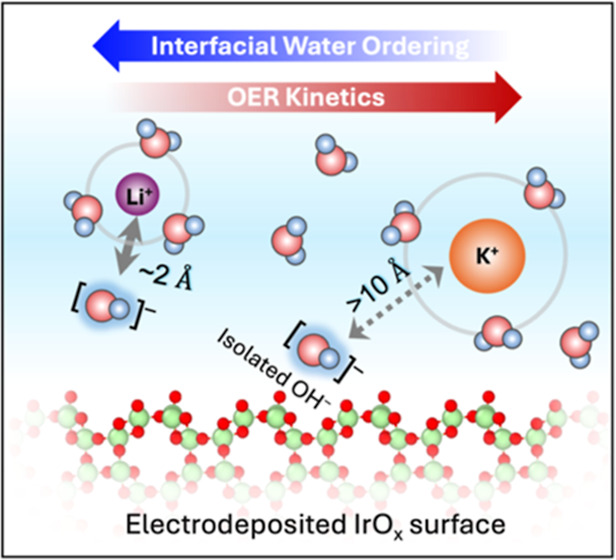

## 1 Introduction

Hydrogen is vital in achieving a net-zero society; its only combustion product is water, and it has a high energy density of around 120 kJ/g, which is three times that of conventional fossil fuels like gasoline.^1^ Low-temperature water splitting is a scalable method to produce green hydrogen.^1^ However, it suffers from the sluggish kinetics of the oxygen evolution reaction (OER) at the anode as it involves successive steps; among which the O–O bond formation step is generally considered the rate-determining step for iridium-based catalysts, which are currently the most effective materials for OER under acidic conditions.^2–5^ Extensive research has been devoted to materials screening for improved OER catalysts, which has relied on correlating OER activities with the binding energies of the intermediates (i.e., *OH, *O, *OOH) on the catalyst surface.^2,3^ Due to the scaling relationships between the adsorption energies of oxygenated intermediates and the constant difference between bindings of *OH and *OOH, the theoretical overpotential can be plotted as a function of a single descriptor (ΔG_*O_ – ΔG_*OH_), which gives rise to a volcano plot,^2,3^ where the catalyst at the top of the volcano has optimal binding energetics. The volcano plot is a milestone in the electrolysis field, paving the way for finding potential active catalysts by predicting the OER activity using a single descriptor.

Although such computational studies have enabled screening activity of a large number of materials, they do not capture the intricacies of the electrochemical double layer. Recent literature for a number of electrochemical reactions, such as the hydrogen evolution/oxidation reaction,^6–10^ the oxygen evolution/reduction reaction,^11–14^ and the CO_2_ reduction reaction^15–18^ among others, have reported that electrolytes are not always innocent, and the influence of pH and alkali metal cations on the activity and selectivity cannot be ignored. OER activity has been widely reported to be dependent on the nature of alkali metal cations. Our previous work showed a decrease in OER activities on rutile RuO_2_(110) when changing the electrolyte from 0.1 M KOH to NaOH and then to LiOH, with the same concentration.^19^ A similar activity trend was observed on Ni­(Fe)­OOH, layered MOF-derived NiFe­(OOH), and CoOOH.^20–22^ The relationship between cation size and OER activity was also demonstrated on NiOOH using alkali metal cations and alkaline earth metal cations, with higher activities observed for larger cations.^23^ On the other hand, Görlin et al. found a partially contradicting trend of Cs^+^ > Na^+^ ≈ K^+^ > Rb^+^ > Li^+^ on electrodeposited NiFe­(OOH), where they proposed that the indirect pH effect due to the different nature of cations was dominant.^24^ Recent work by van der Heijden et al. has further highlighted the influence of cations on water oxidation activity using a combination of cations in the electrolyte.^25^

Several hypotheses have been proposed to explain the cation-dependent activity. Cations can be nonspecifically adsorbed,^11,19,26,27^ specifically adsorbed^20,28–30^ or even intercalated^23,25^ into electrode materials in the case of layered materials. Direct adsorption can change the binding energetics. Crystal truncation rod studies on RuO_2_(110) have shown that cations do not specifically adsorb on oxide surfaces at OER-relevant potentials.^19^ Both the pH and the presence of ions in the electrolyte have been shown to alter the nature of interfacial water at the polarized electrode surface, which can alter the energetics of key intermediates. Our recent work on pH-dependent OER kinetics on amorphous IrO_*x*_ demonstrated the presence of more cation-coordinated water at pH = 13 compared to pH = 1.^31^ With the aid of operando optical spectroscopy, we determined that the energetics for *O binding were stronger at high pHs due to stabilization by polarized water molecules within the hydration shell of the cation. However, the binding energetics were much more strongly dependent on coverage compared to acid due to the steric hindrance limiting the number of hydrated cations at the interface that can stabilize *O species at high coverage. The potential of zero charge (PZC) provides insight into the structure of the interfacial water network, indicating the electrode potential at which the net surface charge is zero and the water dipole orientation is minimized. While it is sensitive to differences in water structuring at the interface, it does not directly reflect variations in specific adsorption or binding interactions between the electrolyte and the electrode surface. Techniques such as laser-induced current transients (LICT) can be used to determine the PZC and the potential of maximum entropy (PME) by tracking the relaxation behavior of interfacial water dipoles in response to thermal perturbations.^32^ Electrolyte cations influence the OER activity through multiple interconnected mechanisms, including modifications to hydration shells, interfacial water structure, local OH^–^ activity, ionic mobility, and the electric double layer. A comprehensive view of polarized solid–liquid interfaces in the presence of different cations, combining the energetics of intermediates and the influence of the double-layer structure on OER kinetics, is missing.

In this work, we investigate the cation-dependent oxygen evolution reaction (OER) on electrodeposited iridium oxide in 0.1 M MOH solutions (M = Li^+^, Na^+^, K^+^, TMA^+^) using a range of operando optical spectroscopy, LICT, surface-enhanced infrared spectroscopy (SEIRAS), and quantum mechanics/molecular mechanics (QM/MM) simulations. We find that the OER activity trend follows: TMA^+^ > K^+^ > Na^+^ > Li^+^. Operando optical spectroscopy was used to quantify the energetics and density of redox species as well as inter-site interactions. Our results show three similar redox transitions in all electrolytes, with similar energetics and densities of oxidized states. LICT results demonstrate a decreasing potential of maximum entropy (PME) from TMA^+^ to K^+^, Na^+^, and Li^+^, suggesting that at OER potentials, the interfacial water layer in the presence of Li^+^ is the most rigid, while for TMA^+^, it is the most disordered. These findings are corroborated by SEIRAS and QM/MM simulations, which show more cation-coordinated water at the interface in the presence of larger cations that can potentially disrupt a well-ordered ice-like water layer, which dominates in the presence of smaller cations. A more disrupted water network at the interface in the presence of larger cations thus facilitates interfacial reorganization during the rate-determining step of the formation of the O–O bond formation. Although IrO_*x*_ is not a state-of-the-art catalyst for alkaline OER, it serves as a well-established model system for fundamental mechanistic studies based on our previous work using optical spectroscopy, X-ray absorption spectroscopy, and DFT calculations on iridium-based catalysts.^5,31,33^ Our work thus provides atomic-level insights into the electrode–electrolyte interface and offers a new, comprehensive understanding of how cations influence the thermodynamics and kinetics of the OER.

## 2 Experimental Methods

### 2.1 Electrodeposition

IrO_*x*_ films were prepared by anodic deposition, which has been reported in previous work by our group, with protocols similar to previous reports.^31,33,34^ The deposition solution was prepared by adding 30 mL of deionized water into a mixture of 0.2 mmol IrCl_3_ hydrate (Fluorochem) and 1 mmol oxalic acid dihydrate (Sigma-Aldrich). Following this, 5 mmol K_2_CO_3_ (Sigma-Aldrich) was added to adjust the pH to ∼10, before adding 20 mL of deionized water to make a 50 mL solution. Then, the mixture was left at 35 °C for 4 days before being stored in a 4 °C refrigerator. A three-electrode setup was used for the IrO_*x*_ deposition, where FTO glass was used as the working electrode, a platinum plate was used as the counter electrode, and Ag/AgCl in saturated KCl (RE-1B, ALS Japan) was used as the reference electrode. The FTO glass was taped with Kapton to limit the deposition area to 1 × 1 cm^2^. Then, it was immersed in approximately 50 mL of iridium chloride solution before running chronopotentiometry under a steady current of 35 μA for 1000 s using a Biologic SP150e potentiostat. After electrodeposition, a blue film was formed on the FTO substrate.

For LICT measurements, the samples were prepared the same way, a three electrode system, with the working electrode being AT-cut quartz crystal wafer electrochemical quartz crystal microbalance (EQCM) electrodes (polycrystalline Pt and Au with the surface area of 1.37 cm^2^, Stanford Research Systems, USA), the counter electrode being a Pt plate, and reference being Ag/AgCl in 3 M KCl (SI analytics) with a double junction. Then, it was immersed in iridium chloride solution before running chronopotentiometry under a steady current of 48.0 μA (35 μA/cm^2^) for 1000 s using a Biologic-VSP300 potentiostat.

For surface-enhanced infrared absorption spectroscopy samples, a Pt-coated Si ATR prism was used as the substrate. The deposition was performed in a similar 3-electrode setup but with a Pt-coated Si ATR prism as the working electrode. Chronopotentiometry of a steady current of 61.6 μA (35 μA/cm^2^) for 1000 s was performed using a Biologic-SP50e potentiostat.

### 2.2 Electrochemical Measurements

The 0.1 M alkaline electrolytes were prepared by dissolving LiOH, NaOH, KOH, and TMAOH (all chemicals from Sigma-Aldrich, 99.995% trace metals basis) in deionized water. TMAOH was used as the bulky cation electrolyte in place of CsOH due to insufficient chemical purity of the available CsOH for sensitive electrochemical and spectroscopic measurements. Electrochemical activity measurements were conducted both with and without an inert atmosphere. An inert environment was established by purging the electrolyte with N_2_ for 30 min prior to the experiment and maintaining N_2_ flow during measurements, with the gas line positioned above the electrolyte to avoid disturbance. A three-electrode setup was used in electrochemistry tests, with the working electrode being a 1 cm^2^ amorphous IrO_*x*_ film deposited on FTO, the counter electrode being a Pt coil (ALS Japan), and the reference electrode being Hg/HgO in 0.1 M KOH (RE-61AP, ALS Japan). The reported potentials were *iR*-corrected with R-values (∼35 Ω) being measured using electrochemical impedance spectroscopy.

### 2.3 Operando Optical UV–Vis Spectroscopy

An operando optical UV–vis spectroscopy protocol has been established by our group.^5,31^ The setup uses a stabilized 10 mW tungsten-halogen light source (SLS201L, Thorlabs), which emits light that passes through a custom-made operando cell with a three-electrode setup. The working electrode was a hydrous IrO_*x*_ film on an FTO substrate, with an area of 1 × 1 cm^2^. The counter electrode was a Pt coil (ALS Japan), and the reference electrode was Hg/HgO in 0.1 M KOH (RE-61AP, ALS Japan). The transmitted light was collected by a liquid light guide with a diameter of 1 cm (Edmund Optics) before refocusing to pair the spectroscope (Kymera 193i, Andor) and a charge-coupled device camera (iDus Du420A-BEX2-DD, Andor). Electrochemical data were collected with an Autolab PGSTAT204 potentiostat. The potentiostatic mode was applied with the current measured at stepped potentials with a 1 mV interval across the entire cyclic voltammetry (CV) range. Custom-built software in LabVIEW recorded the optical and electrochemical signals simultaneously, ensuring they were on the same time scale.

Square-wave measurement was performed by setting a stepped potential profile: V1 for 20 s, then V2 (a more oxidizing potential) for 10 s, before going back to V1 for another 20 s. For each redox transition, the selection of V1 and V2 was within the region where only one intermediate exists. For example, for redox transition 1 in 0.1 M LiOH, the potential window was between 0.506 V_RHE_ and 0.646 V_RHE_, with intermediate potentials having 20 mV increments. Similarly, for redox transition 2, the potential was from 0.906 V_RHE_ to 1.07 V_RHE_, with 20 mV increments; for redox transition 3, the potential was from 1.37 V_RHE_ to 1.51 V_RHE_. The optical absorption was measured by using the spectrometer mentioned above, and the corresponding electrochemistry data were measured by using an Autolab PGSTAT204potentiostat.

Potential decay measurements were performed by holding the system at a potential V1 near the OER region for 20 s, then going to an oxidizing potential V2 for 20 s to form oxidized species before applying open-circuit potential for 80 s more. For example, for 0.1 M KOH, the system was set at 1.406 V_RHE_ for 20 s then at 1.426 V_RHE_ for another 20 s before going to open-circuit potential for 80 s. The whole process was repeated six times while increasing V2 by 20 mV every time; i.e., V2 is 1.426 V_RHE_, 1.446 V_RHE_, 1.466 V_RHE_, 1.486 V_RHE_, and 1.506 V_RHE_. The configuration of the setup remained the same throughout the entire experiment, and optical absorption was recorded using the same spectrometer mentioned above.

### 2.4 LICT Measurements

The laser-induced current transient measurements were performed using a Nd/YAG laser (Spectra-Physics Lasers, USA) operating at 532 nm with a pulse width of 5–8 ns and a repetition rate of 10 Hz, controlled by GCR software. The laser beam had a diameter of ∼9 mm. A motorized variable attenuator (VA-CB-532-CONEX, Newport Corp.), operated via CCVA-PR-CD software (Spectra-Physics Lasers, USA), was used to control the pulse energy density to ∼12.5 mJ cm^–2^ and prevent damage to the working electrode.^21^ Optical alignment was carried out using low-power laser illumination (0.01 W) to ensure normal incidence on the sample through a 30 mm diameter quartz window. The potentiostatic mode was applied at stepped potentials with a 25 mV interval across the whole cyclic voltammetry (CV) range. Measurements were initiated only after the background current stabilized, and the laser was operated in the pulsed mode at 0.1 s intervals for a total duration of 4 s, with a laser power of 0.1 W. For each potential, the transient current was recorded 2 s after laser initiation to avoid artifacts from potential overheating during prolonged irradiation. The current responses due to laser interruptions were recorded with a Biologic-VSP300 potentiostat. Three electrode setup was used during the experiment, with the working electrode being the electrodeposited IrO_*x*_ film on Au/Pt QCM in a QCM holder (Stanford Research Systems, USA), the reference electrode being Hg/HgO (BAS Inc., Japan) with a double junction connected, and the counter being a Pt wire.

### 2.5 Operando Surface-Enhanced Infrared Absorption Spectroscopy (SEIRAS)

The operando SEIRAS measurement protocol has been reported in our previous work.^31^ Pt was first deposited on the total reflecting plane of a hemispherical Si prism (radius 22 mm, Pier optics, Japan) via an electroless deposition method described elsewhere.^35,36^ In short, the surface of the Si hemisphere was given a hydrophilic treatment by polishing with a diamond suspension (1.0 μm, Allied High Tech Products Inc.). After treatment, the surface of the Si prism was immersed in an aqueous 40 wt % NH_4_F solution (98%, Sigma-Aldrich) for 1 min to render the surface hydrophilic. Afterward, the palladium seeds were deposited on the surface by submerging the prism in a mixed aqueous 1 wt % HF solution (48%, Sigma-Aldrich) with 1 mM PdCl_2_ (99%, Sigma-Aldrich) for 5 min at room temperature. After washing the Pd-coated surface with deionized water, the electroless deposition of Pt was conducted by immersing the prism in a Pt plating solution at 50 °C for 5 min. The Pt plating solution was prepared by mixing [Pt­(NH_3_)_6_]­OH_4_ (Tanaka Precious Metal Technologies Co., Ltd.), hydrazine (Sigma-Aldrich), and NH_3_ solution (28%, Sigma-Aldrich).

Operando SEIRAS measurements were performed using a Nicolet iS50 (Thermo Fisher Scientific) instrument equipped with a liquid-nitrogen-cooled HgCdTe (MCT) detector. A three-electrode setup was used with electrodeposited IrO_*x*_ on a Pt-coated Si ATR prism as the working electrode. To remove moisture interference, dry air was fully replaced in the optical path. The resolution for the measurements was 8 cm^–1^. For each condition, 32 scans were averaged. Spectra were recorded by using a custom-made specular reflection unit paired with a Si prism. The incident angle was set to 67°. All spectra are shown in absorbance units defined as log­(*I*_0_/*I*), where *I*_0_ and *I* represent the spectra at the reference and sample potentials, respectively. The reference spectrum, *I*_0_, was measured at 0.6 V_RHE_ in the alkaline electrolytes. The spectra were deconvoluted based on the Gaussian method, where we used the sum of 4 Gaussian peaks to fit the data. We mainly constrained the peak position, which is μ in the following equation for fitting.

$$f\left(x\right)=A\times e^{-{\left(x-\mu \right)}^{2}/2\sigma^{2}}$$

The final peak position (μ) results of fitting were around 3506, 3400, 3226, and 2950 ± 30 cm^–1^ for isolated, asymmetric, ice like, and strongly bond water, respectively. Detailed information on the peak position (μ) and fwhm (σ) can be found in the Supporting Information.

### 2.6 Molecular Simulation Results

Mean-field quantum mechanics/molecular mechanics (QM/MM) multiscale simulation, density functional theory in classical explicit solvents (DFT-CES), is implemented in our in-house code, which combines the Quantum ESPRESSO plane-wave density functional theory (DFT) simulation engine with the LAMMPS molecular dynamics (MD) simulation engine.^37–39^ The amorphous IrO_*x*_ surface is modeled as hollandite IrO_2_ (001), referring to our previous study.^5^ The projector-augmented-wave (PAW) method was used with a kinetic energy cutoff of 50 Ry.^40^ Gaussian smearing was used with a value of 0.2 eV, and the Perdew–Burke–Ernzerhof (PBE) exchange–correlation functional was employed.^41^ A (3 × 3 × 1) Γ-centered *k*-point grid was used to sample the reciprocal space, and a dipole correction along the *z*-direction was applied to block the unphysical interaction between the images of the cells.

The electrolyte phase was modeled using the canonical ensemble MD based on a classical force-field (FF)-type description, and the TIP3P-EW water model was used.^42^ The FF parameters for the cations, hydroxide ions, and IrO_*x*_ are based on the previous studies with the geometry mixing rule.^43–46^ A Nosé–Hoover thermostat was used to maintain the temperature at 300 K, with a damping parameter of 100 fs.^47,48^ Periodic boundary conditions were applied along the *x*- and *y*-directions, and long-range electrostatic interaction in the simulation cells was treated using the modified particle–particle particle–mesh method for slab geometry.^49^

At every DFT-CES iteration, we performed an MD simulation for 6 ns and sampled the last 5 ns trajectory to calculate the average electrostatic potential of the electrolyte phase, which was used in the subsequent DFT calculation as an external potential. During the DFT calculations, only the electronic structure was optimized based on the external potential obtained from the previous MD iteration. The DFT-CES iteration was repeated until the difference in the DFT total energy between the iterations converged below 0.1 kcal mol^–1^. Autocorrelation function of the ion–water pair was determined by identifying ion–water pairs coordinated at a given time step and monitoring their subsequent dissociation.

The computational hydrogen electrode method was used for calculating the Gibbs free energy (Δ*G*) change along the reaction coordinates.^50^ Every Δ*G* is defined as Δ*G* = Δ*E* + ΔZPE – *T*Δ*S* + Δ*G*_sol_, where Δ*E* is the reaction energy difference between the product and reactant, which can be directly calculated by DFT, ZPE is zero-point energy, *T* is the temperature at 298.15 K, and *S* is entropy. For gas molecules, translational, rotational, and vibrational entropies were considered by using particle-in-a-box, rigid rotor, and harmonic oscillator partition functions, respectively. For intermediates adsorbed on the metal, only the vibrational entropy was considered, neglecting the motion of the electrode by using the partial Hessian approach.^51^ Δ*G*_sol_ is a free energy of electrolytes calculated from the two-phase thermodynamics method.^52,53^

## 3 Results and Discussion

### 3.1 Cation-Dependent OER Activity

Film characterization was performed by using SEM and XRD. SEM analysis (Figure S1; see Supporting Information for more details) shows that the as-deposited IrO_*x*_ film uniformly covers the FTO substrate and has a rough, nanogranular morphology. XRD measurements (Figure S2) show no diffraction peaks corresponding to crystalline IrO_2_, confirming that the film is XRD-amorphous. Cyclic voltammograms of electrodeposited IrO_*x*_ films (Figure 1a) show similar features in 0.1 M alkaline solutions with different metal cations, with two distinguishable redox peaks identified at ∼0.65 V_RHE_, ∼1.0 V_RHE_, and a third redox peak emerging at the OER-relevant potentials. In the case of TMA^+^, the first two redox peaks are shifted notably to positive potentials, at 0.70 and 1.10 V_RHE_, respectively. Electrochemical activity measurements were performed with and without an inert atmosphere. Inert conditions were established by purging the electrolyte with N_2_ for 30 min prior to measurements and maintaining the flow of N_2_ above the electrolyte during experiments. The results (Figure S3) show negligible differences between the purged and unpurged conditions across all electrolytes studied. From our previous work, these two redox transitions can be assigned to the deprotonation of *H_2_O on coordinatively unsaturated Ir sites (CUS), followed by the deprotonation of the bridging oxygen.^5,31^ Regardless of the different metal cations (Li^+^, Na^+^, and K^+^), the positions and redox capacitance of the first and second redox peaks remain very similar, which suggests that the coverages of the active sites are independent of metal cations. Similar observations can be made on crystalline IrO_2_ surfaces with lower scan rate, where no distinctive shifts in the first two peak positions is observed.^54^ Nevertheless, the oxygen evolution activity is cation dependent (as shown in Figure 1b), being the highest with TMA^+^, followed by K^+^ and Na^+^, and the lowest in Li^+^; similar to the activity trends on layered NiFe MOF and IrO_2_(110) surfaces.^21,54^ Figure S4 shows the current density as a function of overpotential for the electrolytes described above, highlighting the cation-dependent differences in the OER activity.

**Figure 1:**
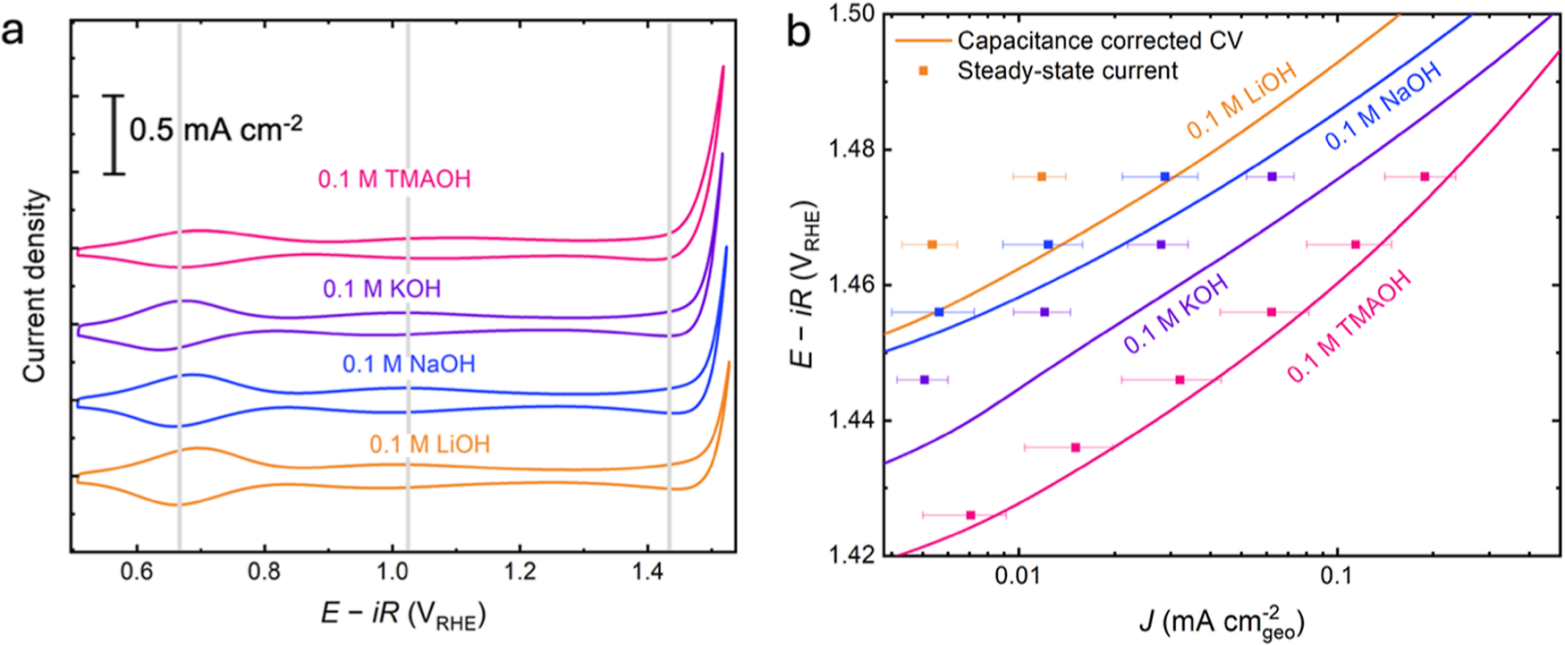
(a) Cyclic voltammograms of electrodeposited IrO_*x*_ films on FTO substrates, at 10 mV/s in 0.1 M TMAOH (pink), 0.1 M KOH (purple), 0.1 M NaOH (blue), 0.1 M LiOH (orange) under room temperature, with a Pt coil as the counter and the Hg/HgO in 1 M KOH as the reference electrode. (b) OER activity measurements: capacitance-corrected cyclic voltammograms (in lines) and steady-state current (in squares) in 0.1 M TMAOH, 0.1 M KOH, 0.1 M NaOH, 0.1 M LiOH. Steady-state currents were determined by averaging chronoamperometric measurements obtained from three individual measurements at the specified potentials; error bars represent the standard deviation.

### 3.2 Potential-Dependent Energetics and Kinetics of Redox-Active Species

To quantify the changes in redox active species as a function of potential and determine the thermodynamic free energies of binding for surface intermediates, we employed operando optical UV–vis (UV–vis) spectroscopy. Figure 2a shows the optical absorption spectra in 0.1 M KOH from 0.5 V_RHE_–1.5 V_RHE_ in 1 mV steps, with the spectra at 0.5 V_RHE_ used as a reference. Three distinctive absorption features could be identified at 650 nm, 800 nm, and 500 nm with increasing potential. The corresponding potential regions for these features are ∼0.5 V_RHE_ to ∼0.8 V_RHE_, ∼0.8 V_RHE_ to ∼1.1 V_RHE_, and >1.1 V_RHE_, which agrees with our previous findings.^31^ Similarly, the optical spectra in 0.1 M LiOH (Figure 2b) show similar features in this potential range. These findings indicate that the same three redox species were generated at similar potentials. Likewise, in 0.1 M TMAOH and 0.1 M NaOH, comparable optical absorption spectra were observed (Figure S5), with the same three features appearing in the corresponding potential regions, further suggesting consistent redox behavior across different alkaline electrolytes. We have assigned these three redox transitions to deprotonation of *H_2_O and *H from surface coordinatively unsaturated (CUS) and bridge oxygen sites, based on detailed analysis in our previous work^5,31^

redoxtransition1:H*2O(cus)+OH−→O*H(cus)+H2O+e−

redoxtransition2:O*H(cus)+*Hb+OH−→O*H(cus)+H2O+e−+*

redoxtransition3:O*H(cus)+OH−→O*(cus)+H2O+e−

**Figure 2:**
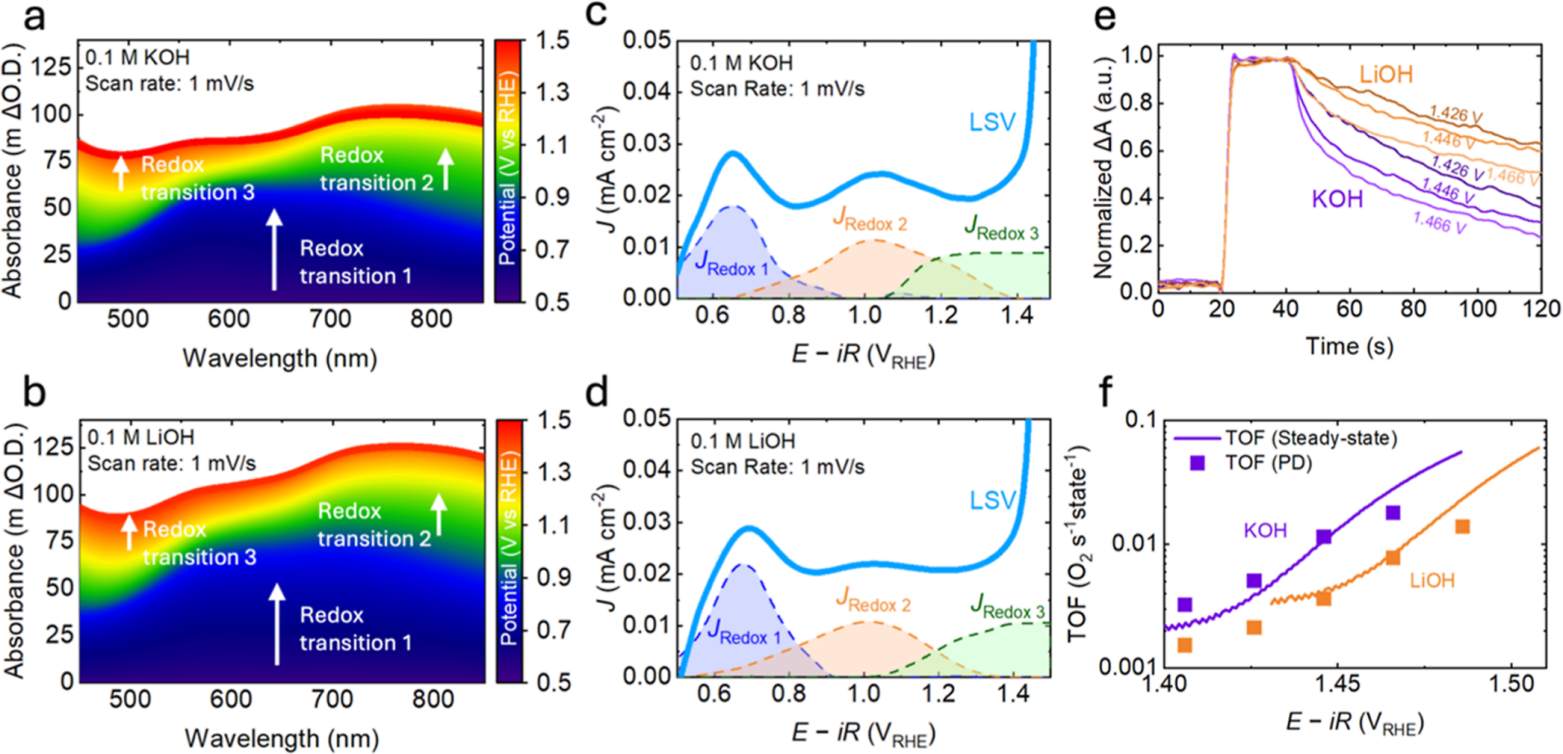
(a,b) Differential optical absorption spectra for 0.1 M (a) KOH, (b) LiOH during a linear sweep from 0.5 V_RHE_ to 1.5 V_RHE_, at a scan rate of 1 mV/s. (c,d) Charging currents (dashed lines) of redox transition 1­(blue), 2 (orange), 3 (green) plotted as a function of potential, deconvoluted from optical spectra, in 0.1 M (c) KOH, (d) LiOH; the areas under these dotted lines indicate the charges transferred during these processes. The electrochemical current signal is shown in a solid light-blue line. (e) Comparison between normalized absorption spectra from potential decay measurements, where the system was held at 1.386 V_RHE_ for 20 s, then held at more oxidizing potentials (as indicated) for another 20 s, before it was put under open circuit for a further 80 s. The results for KOH are shown in purple, while those for LiOH are shown in orange. Data were smoothed using the Lowess method with a window size of around 200 points. (f) Turnover frequency (TOF) as a function of potential for 0.1 M LiOH (orange) and 0.1 M KOH (purple), calculated in 2 different ways. TOF calculated from potential decay measurements are shown in points, while from steady-state currents are shown in solid lines.

To quantify the potential-dependent density of these redox species, we have deconvoluted the optical spectra into a linear combination of these three redox species (the deconvolution method has been reported previously^5,31,33^ and can also be found in Supporting Information). Briefly, differential analysis was performed by finding the differences between the adjacent spectra and then plotting the normalized data at a 10 mV interval (Figure S6). Spectra representing individual redox transitions were selected from potential ranges where the normalized differential spectral profiles remained unchanged. This suggests that the observed absorption changes primarily reflect differences in the concentration of the particular species. Three distinctive differential spectra at 0.65–0.66 V_RHE_, 0.98–0.99 V_RHE_, and 1.41–1.42 V_RHE_ were used in the fitting to get simulated differential spectra (Figures S7–S10). The simulated spectra closely matched the experimental data across all electrolytes examined (Figures 2, and S5). Comparisons of the three selected potentials revealed minimal differences, and the residuals from the fittings remained within ±4% error across all electrolytes (Figures S7–S10). These results confirm that the simulated spectra reliably represent the experimental data. The calculated spectra were deconvoluted into redox transitions as a function of potential (Figures S7–S10). Applying the extinction coefficient (Figures S11–S14) converts the optical signal into an electrical current, which can then be expressed as a function of potential.

The fitted redox transitions in Figure 2c,d for 0.1 M KOH and 0.1 M LiOH and in Figure S5 for TMAOH and NaOH with the same concentration enable us to compare the energetics of the three redox transitions in the presence of different electrolytes. For 0.1 M LiOH, 0.1 M NaOH, and 0.1 M KOH, a similar value of U_(θ=1/2)_ for redox 1 (U_(θ=1/2)_ LiOH = 0.68 V_RHE_, U_(θ=1/2)_ NaOH = 0.68 V_RHE_, U_(θ=1/2)_ KOH = 0.66 V_RHE_, U_(θ=1/2)_ TMAOH = 0.72 V_RHE_), redox 2 (U_(θ=1/2)_ LiOH = 0.98 V_RHE_, U_(θ=1/2)_ NaOH = 1.04 V_RHE_, U_(θ=1/2)_ KOH = 1.04 V_RHE_, U_(θ=1/2)_ TMAOH = 1.10 V_RHE_), and redox 3 (U_(θ=1/2)_ LiOH = 1.41 V_RHE_, U_(θ=1/2)_ NaOH = 1.33 V_RHE_, U_(θ=1/2)_ KOH = 1.38 V_RHE_, U_(θ=1/2)_ TMAOH = 1.39 V_RHE_) is observed (Figure S15). For 0.1 M TMAOH, redox 1 and 2 exhibit slightly anodic shifts to 0.72 and 1.10 V_RHE_ for redox 1 and 2, but U_(θ=1/2)_ for redox 3 remains unaltered. Notably, the electroadsorption isotherms obtained using optical spectroscopy could not be fitted to a Langmuir model based on the assumption of no interaction between neighboring sites. Instead, we employed Frumkin isotherm fitting (see Supplementary Note for more information). The *r* parameters, describing intersite interactions, are 0.37 for 0.1 M KOH, 0.35 for LiOH, 0.33 for NaOH, and 0.27 for TMAOH. The similar *r* values for K^+^, Li^+^, and Na^+^ suggest comparable lateral repulsive interactions between adsorbed species on the IrO_*x*_ surface. The slightly lower *r* value for TMA^+^ indicates weaker repulsion, possibly due to its larger ionic radius and lower surface charge density. The interaction between surface adsorbates on IrO_*x*_ has been hypothesized to occur via the interfacial electrolyte.^4^ Notably, in our previous work in 0.1 M HClO_4_, we found a markedly different interaction parameter of around 0.13 eV, which we attributed to the difference in interfacial water structure as a function of pH.^31^ Here, interestingly, we find that altering the nature of cations in the electrolyte does not have a strong influence on the binding energetics or intersite interactions.

Although similar binding energetics and intersite interactions were found in all electrolytes, the intrinsic activity for the OER was strongly cation-dependent, as probed using optical spectroscopy. Two independent ways were used to assess the rate of turnover: one from steady-state current measurements and the other from potential decay measurements. First, we obtained the turnover frequency, defined as the number of oxygen molecules generated per oxidized species, by normalizing the OER current to the density of oxidized species. Considering that the OER current is significantly larger for larger cations (Figure 1b), but the density of oxidized species is cation-independent (Figure 2c,d), we determine that the intrinsic TOF is strongly dependent on the nature of the cation (Figure 2f). These results are further supported by open-circuit decay measurements.^55^ In these measurements, first, the electrode is held at potential V1 for 20 s, before being held at a more oxidizing potential, V2, for a further 20 s. Following this, the circuit is switched to an open circuit. The optical absorption increases as more redox species are generated when increasing the potential to V2 and decreases under open-circuit conditions. Figure 2e shows a comparison of normalized absorption spectra from potential decay measurements for 0.1 M KOH and LiOH, where the initial rate of decay provides insights into how fast the oxidized species are consumed to drive the OER. For the same applied potentials and consequently the same density of oxidized species, faster turnover is observed in 0.1 M KOH compared to LiOH (Figure 2f), consistent with our findings from steady-state current measurements. With larger cations, faster decay is observed (Figure S16), which means they have a faster turnover (Figure S17). In summary, from optical spectroscopy, we find 3 similar redox reactions with approximately the same population and energetics of redox-active species. However, we determine that the intrinsic kinetics are markedly different and cannot solely be described by different binding energetics or intersite interactions. These findings thus motivate the study of the interfacial solvent structure at OER-relevant potentials in the presence of different electrolytes.

### 3.3 Probing the Interfacial Water Structure

To probe the electrode–electrolyte interface, the laser-induced current transient technique was employed. A short laser pulse locally heats the double layer region, causing the reorientation of interfacial water dipoles due to thermal excitation. When the laser is turned off after 0.1 s, the water molecules relax back to their initial states, generating a transient current. This current reflects changes in surface charge density with higher interfacial polarization, leading to more ordered water dipoles and a stronger transient signal. The potential of maximum entropy occurs when the current response is zero, indicating that the interface is in its most chaotic state. For 0.1 M LiOH, the current response was recorded against time and applied potential (Figure 3a). At lower potentials, from 0.7 V_RHE_ to 1.0 V_RHE_, the induced current is negative, suggesting that the surface carries a negative charge and water molecules are oriented with the hydrogen atom pointing toward the surface; while at potentials higher than 1.15 V_RHE_, the current response is positive, showing a positively charged interface. As the potential becomes more oxidizing, the induced current becomes more positive. Figure 3a shows the excess charges on the electrode surface, and we can see a clear transition from negative (shown in black lines) to positive signals (shown in orange) at a potential between 1.05 V_RHE_ and 1.10 V_RHE_ in 0.1 M LiOH. The charge density (Figure 3b), obtained by integrating the current signals over time from Figure 3a, crosses 0 at 1.09 V_RHE_, indicating that this value corresponds to the PME in 0.1 M LiOH. Similarly, we can identify the PME for 0.1 M KOH as 1.15 V_RHE_ from the 2D charge density profile (Figure 3c). The PME value was further confirmed through current density calculations, which produced identical results (Figure S18). The PME is strongly cation dependent, with values ranging from 1.26 V_RHE_ for 0.1 M TMAOH (Figure S19), 1.15 V_RHE_ for 0.1 M KOH, 1.12 V_RHE_ for 0.1 M NaOH (Figure S19), and 1.09 V_RHE_ for 0.1 M LiOH, which suggests a strong dependence of PME on the nature of cations, agreeing with previous studies on NiFe­(OOH) MOF electrocatalysts and Pt(111).^21,56^ Moreover, a linear correlation between OER activity and PME is observed (Figure S20), in agreement with trends reported in earlier studies on NiFe­(OOH) MOF systems, further supporting the generality of this relationship.^21^ The PME for larger cations is closer to the OER-relevant potentials, implying that the solid/liquid interface is less ordered in the presence of larger cations. Therefore, we postulate that larger cations induce weaker ordering of water molecules at OER conditions, which facilitates charge transfer and O–O bond formation via dissociation of a water molecule on *O species.^31,57^

**Figure 3:**
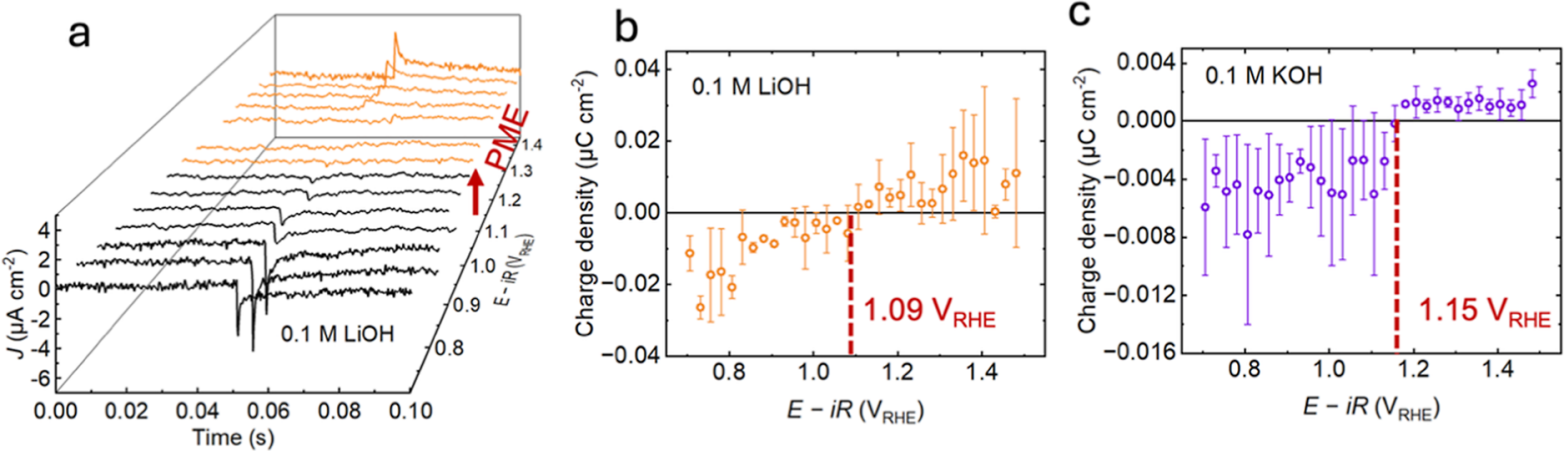
(a) 3D plots of current transient signals due to pulsed laser within the potential range from 0.7 V_RHE_ to 1.5 V_RHE_ at 25 mV intervals for 0.1 M LiOH. The negative current responses are indicated by black lines while the positive responses are shown in orange; potential of maximum entropy (PME) could be found when induced current is 0, indicated by the red arrow; (b,c) 2D LICT data showing the correlation between charge density and applied potential for (b) 0.1 M LiOH, (c) 0.1 M KOH. Charge densities are averaged from forward (oxidizing) and backward (reducing) scans; error bars denote standard deviation. PMEs have been indicated using red dashed lines: 1.09 V_RHE_ in 0.1 M LiOH, 1.15 V_RHE_ in 0.1 M KOH. Both experiments used Pt wire as the counter electrode and Hg/HgO as the reference electrode.

In order to rationalize the differences in the degree of ordering observed for the different electrolytes, we performed operando surface-enhanced infrared absorption spectroscopy (SEIRAS) to determine the nature of the interfacial water molecules. A three-electrode setup with electrodeposited IrO_*x*_ on a Pt-coated Si ATR prism was used for the SEIRAS. CV (Figure S21) and spectral comparisons (Figure S22) with bare Pt confirm complete IrO_*x*_ coverage, making it the main contributor to the electrochemical and SEIRAS signals. Figure 4a,b shows the SEIRAS spectra for the O–H stretching modes (2500–4000 cm^–1^) at different potentials in 0.1 M LiOH and KOH, respectively. Similar features were found as a function of potential, with increasing intensity at higher applied potentials (Figure S23). As shown in Figure 4c, the spectra can be deconvoluted into 4 types of H-bonding environments, consistent with previous reports,^58–61^ i.e., (1) isolated OH groups, including either OH^–^ ions or water molecules which have no hydrogen bonding and does not interact strongly with the surrounding environment, having a peak centered at 3580 cm^–1^;^60,62,63^ (2) asymmetric water or OH^–^ species (peak centered at 3400 cm^–1^), which have incomplete H-bonding network with 1 to 3 hydrogen bonds and can correspond to water/OH^–^ within the hydration shell of cations;^6,19,63^ (3) ice-like water or OH^–^ with peak at 3200 cm^–1^, which has symmetrical hydrogen bonds, with 4 hydrogen bonds per water molecule, similar to bulk water;^19,61^ and (4) strongly bonded water with peak at the lowest wavenumber of 2900 cm^–1^, and is assigned to the water in the hydrated structure of IrO_*x*_.^19,64,65^ We note that previous reports have suggested that the feature at 3580 cm^–1^ can also be attributed to surface-bound *OH species.^66^ However, based on our previous studies combining X-ray absorption spectroscopy and density functional theory,^5^ we expect the density of surface-bound *OH groups to be greatest after redox transition 1, with their deprotonation occurring only at higher potentials. Considering the species at 3580 cm^–1^ in the SEIRAS spectra does not follow this potential dependence (Figures S24 and S25), we attribute this feature to arise from the electrolyte as opposed to surface-bound intermediates. Detailed information on the fitting parameters like peak position and full width at half-maximum (fwhm) for each water species could be found in the Supporting Information (Tables S1–S4). As shown in Figure 4d, the interfacial water at 1.6 V_RHE_ in 0.1 M LiOH is dominated by strongly bonded water and ice-like water. With increasing cation size to 0.1 M KOH, the fractions of isolated and asymmetric water increase. A similar trend is observed with 0.1 M NaOH and TMAOH (Figures S24 and S25). While the overall water content remains relatively constant from 1.0 to 1.6 V_RHE_, the abundance of isolated and asymmetric water species increases notably with increasing cation size, from Li^+^ to Na^+^, K^+^ and TMA^+^ under the same potential. The increase in the asymmetric water species with increasing cation size suggests the presence of more cation-coordinated water near the interfaces, implying that larger cations disrupt ordered water layers near the interface. This hypothesis also supports the presence of more isolated water molecules at the interface in the presence of larger cations, unlike the larger fraction of 4-fold coordinated ice-like water that is present at the interface in the presence of smaller cations such as Li^+^. This result is also in line with LICT analysis (Figure S26), which showed that near the OER potentials, the interfacial water is more disordered in the presence of larger cations.

**Figure 4:**
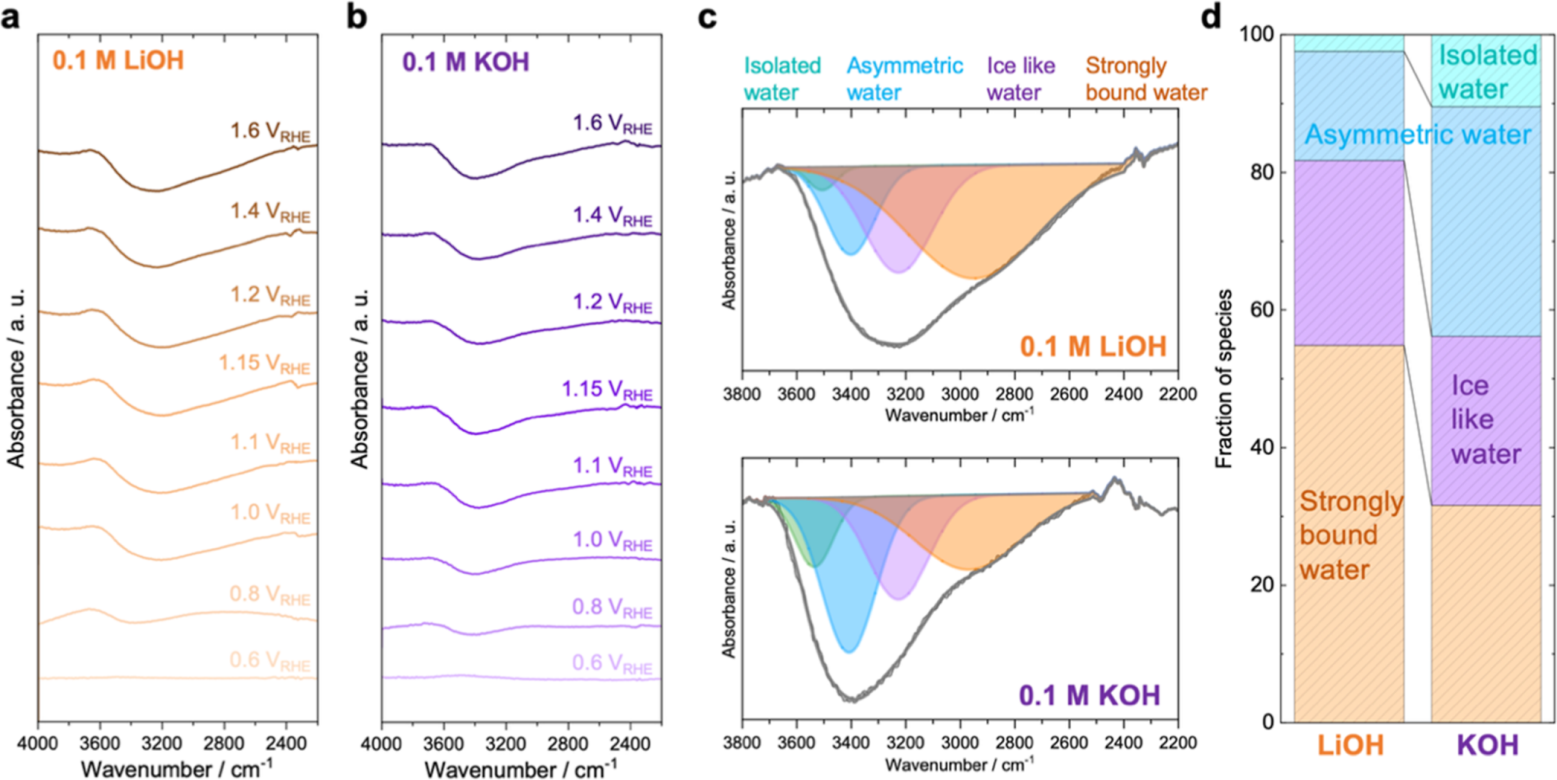
(a,b) Operando SEIRA spectra in the O–H stretching regime, under different applied potentials with the spectra at 0.6 V_RHE_ as reference, in (a) 0.1 M LiOH, (b) 0.1 M KOH; platinum plate and Ag/AgCl were used as CE and RE, respectively, for the SEIRAS measurement. The baseline of each spectrum was corrected using OMNIC software with a three-point autocorrection method. (c,d) Deconvolution of the O–H stretching vibration peak at 1.6 V_RHE_ in 0.1 M LiOH (top) and 0.1 M KOH (bottom) solutions. (d) Quantification of interfacial water structures at 1.6 V_RHE_.

Further support for the nature of interfacial water at relevant potentials of the OER in the presence of different electrolyte environments comes from QM/MM simulations. The simulation setup consists of IrO_*x*_ surface (QM region) with KOH and LiOH electrolyte (MM region) that are electrostatically coupled (Figure 5a,b). The governing equations for the electrode and electrolyte are DFT and classical mechanics, respectively, with a bespoke interfacial Hamiltonian.^37^ Notably, the free energy difference using different electrolytes showed a marginal difference in the energetics of the four consecutive deprotonation steps, with different cations (Figure S27), consistent with our optical spectroscopy results. The theoretical overpotential is ∼0.3 V for both K^+^ and Li^+^ electrolytes, which is well aligned to the experimental findings. Figure 5a,b demonstrates that OH^–^ in the presence of LiOH are in close proximity to the cation (Li^+^–OH^–^ distance ∼2 Å), whereas they are weakly interacting with the larger K^+^ cation (K^+^–OH^–^ distance ∼10 Å). This aligns with the SEIRAS results, which suggest the presence of more isolated OH^–^ groups at the interface for larger cations, which can react more readily with surface *O groups to form the O–O bond. At equilibrium, the radial distributions of cations and OH^–^ are calculated at this interface (Figure 5c). A dominant peak is observed in the case of LiOH at ∼2 Å, suggesting a more ordered interface. On the contrary, the interfacial structure in the case of 0.1 M KOH does not exhibit strong ordering, as evidenced by the lack of features in the radial distribution plot, supporting the notion that larger cations disrupt the water network at the interface. Finally, the water coordination to the cations and its autocorrelation function (ACF) are calculated (Figure 5d). The ACF with K^+^ decays under 100 ps, but the ACF with Li^+^ has an order of magnitude larger time scale, i.e., in ns. This further indicates that cations with more compact hydration shells, such as LiOH, have more well-ordered water molecules at the interface, whereas larger cations such K^+^ interact weakly with water molecules within their hydration shell and thus create a disrupted water network at the interface. A disrupted solvent environment, facilitating the presence of isolated and reactive OH^–^ at the interface, is key to enabling the formation of an O–O bond. Although this study focuses on 0.1 M electrolytes, the insights can be extended to more concentrated solutions to explore changes in the interfacial layer. Increasing overall molarity also changes the pH, which in alkaline media increases the negative surface charge, strengthens the interfacial electric field, and stiffens the water layer by distorting dipoles and disrupting hydrogen bonding.^6,68,69^ Alternatively, adjusting the concentration of a specific cation while compensating with another to maintain constant pH introduces mixed-cation effects, complicating the relationship with PME.^27,70^ Consequently, whether a well-defined cation concentration threshold exists for the observed trend remains an open question, with current evidence suggesting a more continuous rather than threshold-like dependence.

**Figure 5:**
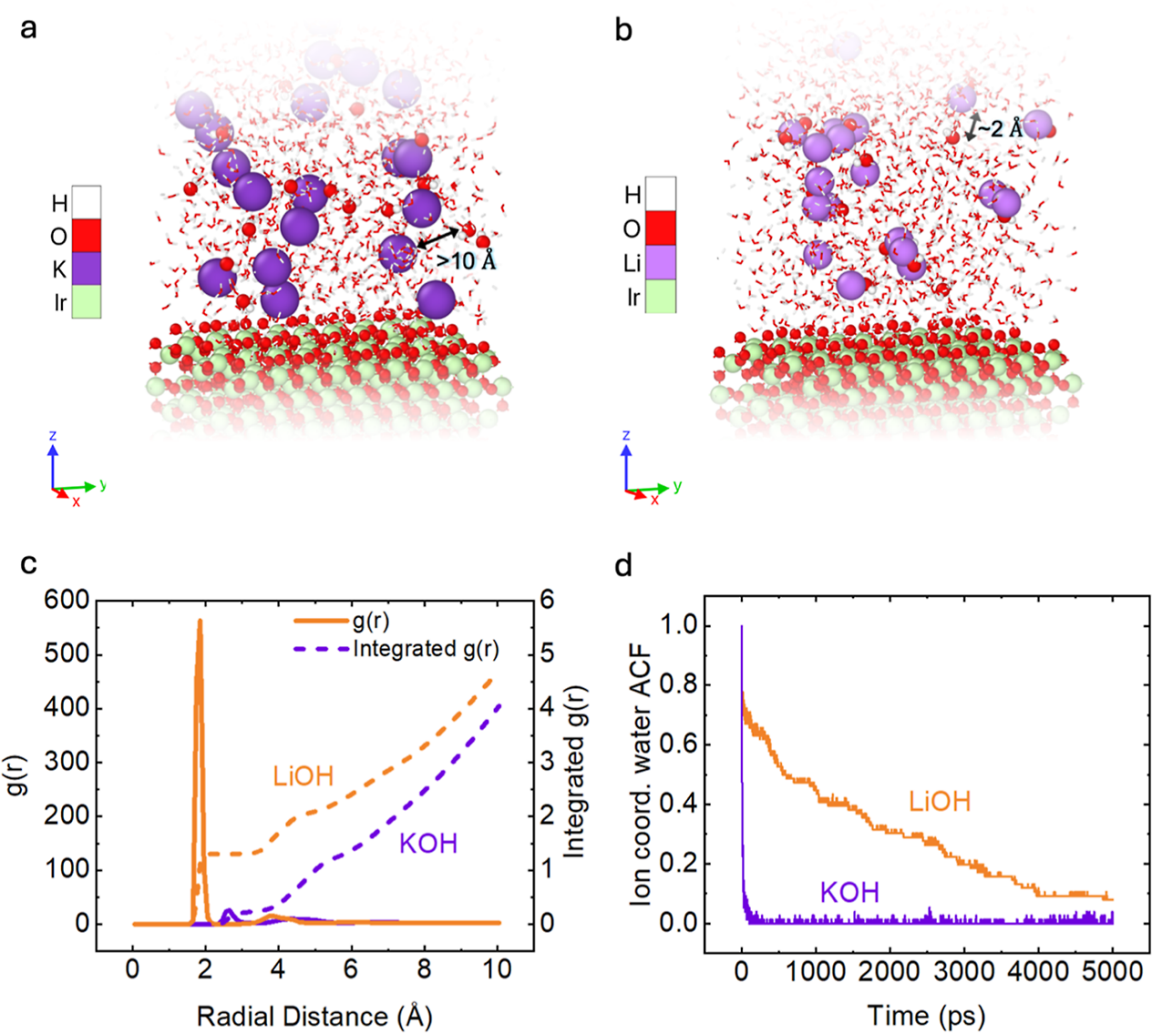
Atomic-scale models for electrochemical QM/MM simulations, for (a) KOH and (b) LiOH, where the iridium oxide surface is described quantum mechanically and electrostatically coupled to the electrolyte. (c) Radial distribution function, *g*(r), with cations and hydroxide ions. (d) Autocorrelation function of ion coordinated water with different electrolyte conditions.^3,67^

From the discussion above, we observe that although optical spectroelectrochemical measurements indicate no significant change in adsorbate binding or inter-site interactions, LICT and vibrational spectroscopy, supported by QM/MM calculations, reveal changes in the potential of maximum entropy and the structure of the interfacial water network. We acknowledge that electrolyte cations influence OER activity through multiple interconnected mechanisms, including modifications to hydration shells, interfacial water structure, local OH^–^ activity, ionic mobility, and the electric double layer.^19,21,24,56,68^ However, our results suggest that the dominant effect in this case results from the structure of the interfacial water network. These findings underscore the importance of employing complementary techniques to gain a comprehensive understanding of complex electrochemical interfaces. Collectively, our results highlight that electrolyte cations play an active role in modulating the interfacial environment and catalytic performance—extending beyond their conventional role as inert spectators. Consistent with prior studies reporting enhanced OER rates with larger cations across a wide range of catalysts, including crystalline RuO_2_, Ni­(Fe)­OOH, layered MOF-derived NiFe­(OOH), and CoOOH, our observations suggest that the underlying interfacial mechanisms identified here are broadly applicable.^19–22,24^ As such, these insights are likely transferable to other electrode–electrolyte interfaces, with implications that extend far beyond iridium-based oxide systems, and offer valuable insights for the rational design of electrolyte–electrode interfaces.

## 4 Conclusions

This work uses a combination of operando optical spectroscopy, LICT, SEIRAS, and QM/MM simulation to investigate the physical origin of cation-dependent OER kinetics. We observed a decrease in the OER activity from TMA^+^ to K^+^, Na^+^, and Li^+^. From operando optical spectroscopy, three similar redox transitions were identified within the potential range from 0.5 to 1.5 V_RHE_. The final redox transition, i.e., formation of *O species, has similar onsets at ∼1.1 V_RHE_ and a comparable number of redox-active species concentration (∼2.0 × 10^16^ cm^–2^), regardless of the cation. However, the intrinsic activity of the OER showed a strong cation dependence, where a higher turnover rate is observed with larger cations. This could be explained by the differences in water structures and the degree of ordering in the interfacial water layer. LICT results revealed that the electrode/electrolyte interface was more disordered with larger cations like K^+^ because the PME got closer to OER onset potentials, making it easier for intermediates to react with absorbed species on the catalyst surface. Operando SEIRAS provided further evidence by quantifying the fractions of various water structures from the O–H stretching regions and found more asymmetric and isolated water/OH^–^ ions at the interface as the size of the cation got larger. We propose that the lower charge density of larger cations has less stabilizing power to interfacial OH^–^, making it easier to react with redox active species on the interface. QM/MM simulations elucidate the detailed interfacial structure and further support the experimental findings by showing a marginal change of thermodynamics with different cations. Instead, it showed the presence of a more ordered solvent environment, with strongly coordinated OH^–^ ions for smaller cations such as Li^+^, which reduced their reactivity with *O to form the critical O–O bond. Therefore, through our work, using multimodal operando characterization and theoretical techniques, we demonstrate the physical origin of cation-dependent OER kinetics, thus demonstrating how tuning the structure of interfacial water and cations provides an additional lever for tuning catalytic activity at complex interfaces.

## Supplementary Material



## Notes

Additional experimental details and methods—including operando optical spectroscopy deconvolution, Frumkin isotherm fitting, complementary LICT and ATR-SEIRAS data, and simulation results (PDF)

## References

[1] Ji M., Wang J. (2021). Review and Comparison of Various Hydrogen Production Methods Based
on Costs and Life Cycle Impact Assessment Indicators. Int. J. Hydrogen Energy.

[2] Man I. C., Su H. Y., Calle-Vallejo F., Hansen H. A., Martínez J. I., Inoglu N. G., Kitchin J., Jaramillo T. F., Nørskov J. K., Rossmeisl J. (2011). Universality
in Oxygen Evolution
Electrocatalysis on Oxide Surfaces. ChemCatChem.

[3] Rossmeisl J., Qu Z. W., Zhu H., Kroes G. J., Nørskov J. K. (2007). Electrolysis
of Water on Oxide Surfaces. J. Electroanal.
Chem..

[4] Nong H. N., Falling L. J., Bergmann A., Klingenhof M., Tran H. P., Spöri C., Mom R., Timoshenko J., Zichittella G., Knop-Gericke A., Piccinin S., Pérez-Ramírez J., Cuenya B. R., Schlögl R., Strasser P., Teschner D., Jones T. E. (2020). Key Role of Chemistry versus Bias in Electrocatalytic
Oxygen Evolution. Nature.

[5] Liang C., Rao R. R., Svane K. L., Hadden J. H. L., Moss B., Scott S. B., Sachs M., Murawski J., Frandsen A. M., Riley D. J., Ryan M. P., Rossmeisl J., Durrant J. R., Stephens I. E. L. (2024). Unravelling the
Effects of Active
Site Density and Energetics on the Water Oxidation Activity of Iridium
Oxides. Nat. Catal..

[6] Huang B., Rao R. R., You S., Hpone Myint K., Song Y., Wang Y., Ding W., Giordano L., Zhang Y., Wang T., Muy S., Katayama Y., Grossman J. C., Willard A. P., Xu K., Jiang Y., Shao-Horn Y. (2021). Cation- and PH-Dependent Hydrogen
Evolution and Oxidation
Reaction Kinetics. JACS Au.

[7] Ledezma-Yanez I., Wallace W. D. Z., Sebastián-Pascual P., Climent V., Feliu J. M., Koper M. T. M., Koper M. T. M. (2017). Interfacial
Water
Reorganization as a PH-Dependent Descriptor of the Hydrogen Evolution
Rate on Platinum Electrodes. Nat. Energy.

[8] Li P., Jiang Y., Hu Y., Men Y., Liu Y., Cai W., Chen S. (2022). Hydrogen Bond Network Connectivity in the Electric
Double Layer Dominates the Kinetic PH Effect in Hydrogen Electrocatalysis
on Pt. Nat. Catal..

[9] Khani H., Puente Santiago A. R., He T. (2023). An Interfacial View of Cation Effects
on Electrocatalysis Systems. Angew. Chem., Int.
Ed..

[10] Monteiro M. C. O., Goyal A., Moerland P., Koper M. T. M. (2021). Understanding
Cation Trends for Hydrogen Evolution on Platinum and Gold Electrodes
in Alkaline Media. ACS Catal..

[11] Strmcnik D., Van Der Vliet D. F., Chang K. C., Komanicky V., Kodama K., You H., Stamenkovic V. R., Marković N. M. (2011). Effects of Li+, K+, and Ba2+ Cations on the ORR at
Model and High Surface Area Pt and Au Surfaces in Alkaline Solutions. J. Phys. Chem. Lett..

[12] Ji S. G., Kim M. M., Han M. H., Cho J., Son Y., Kim Y. Y., Jeong J., Kim Z. H., Chae K. H., Oh H. S., Kim H., Choi C. H. (2024). Alkali Metal Cations
Act as Homogeneous Cocatalysts for the Oxygen Reduction Reaction in
Aqueous Electrolytes. Nat. Catal..

[13] Chen B., Wang P., Shin D., Li W., Liu T., Waegele M. M., Wang D. (2025). Effect of Electrolyte
Ions on Iridium
Oxide-Based Water Oxidation Catalysis. ACS Catal..

[14] Arminio-Ravelo J. A., Jensen A. W., Jensen K. D., Quinson J., Escudero-Escribano M. (2019). Electrolyte
Effects on the Electrocatalytic Performance of Iridium-Based Nanoparticles
for Oxygen Evolution in Rotating Disc Electrodes. ChemPhysChem.

[15] Resasco J., Chen L. D., Clark E., Tsai C., Hahn C., Jaramillo T. F., Chan K., Bell A. T. (2017). Promoter Effects
of Alkali Metal Cations on the Electrochemical Reduction of Carbon
Dioxide. J. Am. Chem. Soc..

[16] Shin S. J., Choi H., Ringe S., Won D. H., Oh H. S., Kim D. H., Lee T., Nam D. H., Kim H., Choi C. H. (2022). A Unifying Mechanism for Cation Effect Modulating C1
and C2 Productions from CO2 Electroreduction. Nat. Commun..

[17] Monteiro M. C. O., Dattila F., López N., Koper M. T. M. (2022). The Role of Cation
Acidity on the Competition between Hydrogen Evolution and CO2Reduction
on Gold Electrodes. J. Am. Chem. Soc..

[18] Sebastián-Pascual P., Mezzavilla S., Stephens I. E. L., Escudero-Escribano M. (2019). Structure-Sensitivity
and Electrolyte Effects in CO2 Electroreduction: From Model Studies
to Applications. ChemCatChem.

[19] Rao R. R., Huang B., Katayama Y., Hwang J., Kawaguchi T., Lunger J. R., Peng J., Zhang Y., Morinaga A., Zhou H., You H., Shao-Horn Y. (2021). PH-and Cation-Dependent
Water Oxidation on Rutile RuO2(110). J. Phys.
Chem. C.

[20] Garcia A. C., Touzalin T., Nieuwland C., Perini N., Koper M. T. M. (2019). Enhancement
of Oxygen Evolution Activity of Nickel Oxyhydroxide by Electrolyte
Alkali Cations. Angew. Chem., Int. Ed..

[21] Hou S., Xu L., Ding X., Kluge R. M., Sarpey T. K., Haid R. W., Garlyyev B., Mukherjee S., Warnan J., Koch M., Zhang S., Li W., Bandarenka A. S., Fischer R. A. (2022). Dual In Situ Laser
Techniques Underpin the Role of
Cations in Impacting Electrocatalysts. Angew.
Chem., Int. Ed..

[22] Jia H., Yao N., Yu C., Cong H., Luo W. (2023). Unveiling the Electrolyte
Cations Dependent Kinetics on CoOOH-Catalyzed Oxygen Evolution Reaction. Angew. Chem., Int. Ed..

[23] Zaffran J., Stevens M. B., Trang C. D. M., Nagli M., Shehadeh M., Boettcher S. W., Caspary Toroker M. (2017). Influence of Electrolyte Cations
on Ni­(Fe)­OOH Catalyzed Oxygen Evolution Reaction. Chem. Mater..

[24] Görlin M., Halldin Stenlid J., Koroidov S., Wang H. Y., Börner M., Shipilin M., Kalinko A., Murzin V., Safonova O. V., Nachtegaal M., Uheida A., Dutta J., Bauer M., Nilsson A., Diaz-Morales O. (2020). Key Activity Descriptors of Nickel-Iron
Oxygen Evolution Electrocatalysts in the Presence of Alkali Metal
Cations. Nat. Commun..

[25] van
der Heijden O., Eggebeen J. J. J., Trzesniowski H., Deka N., Golnak R., Xiao J., van Rijn M., Mom R. V., Koper M. T. M. (2024). Li+ Cations Activate NiFeOOH for
Oxygen Evolution in Sodium and Potassium Hydroxide. Angew. Chem., Int. Ed..

[26] Zhang H., Raciti D., Hall A. S. (2025). Disordered Interfacial H2O Promotes
Electrochemical C–C Coupling. Nat. Chem..

[27] Huang J., Li M., Eslamibidgoli M. J., Eikerling M., Groß A. (2021). Cation Overcrowding Effect on the
Oxygen Evolution
Reaction. JACS Au.

[28] Yang C., Fontaine O., Tarascon J., Grimaud A., ang D. Y., Tarascon J., Fontaine O. (2017). Chemical Recognition
of Active Oxygen
Species on the Surface of Oxygen Evolution Reaction Electrocatalysts. Angew. Chem., Int. Ed..

[29] Mills J. N., McCrum I. T., Janik M. J. (2014). Alkali Cation Specific Adsorption
onto Fcc(111) Transition Metal Electrodes. Phys.
Chem. Chem. Phys..

[30] Goyal A., Koper M. T. M. (2021). The Interrelated Effect of Cations and Electrolyte
PH on the Hydrogen Evolution Reaction on Gold Electrodes in Alkaline
Media. Angew. Chem., Int. Ed..

[31] Liang C., Katayama Y., Tao Y., Morinaga A., Moss B., Celorrio V., Ryan M., Stephens I. E. L., Durrant J. R., Rao R. R. (2024). Role of Electrolyte
PH on Water Oxidation for Iridium
Oxides. J. Am. Chem. Soc..

[32] Xu P., von Rueden A. D., Schimmenti R., Mavrikakis M., Suntivich J. (2023). Optical Method for Quantifying the
Potential of Zero
Charge at the Platinum–Water Electrochemical Interface. Nat. Mater..

[33] Bozal-Ginesta C., Rao R. R., Mesa C. A., Liu X., Hillman S. A. J., Stephens I. E. L., Durrant J. R. (2021). Redox-State Kinetics
in Water-Oxidation IrOx Electrocatalysts Measured by Operando Spectroelectrochemistry. ACS Catal..

[34] Petit M. A., Plichon V. (1998). Anodic Electrodeposition of Iridium
Oxide Films. J. Electroanal. Chem..

[35] Katayama Y., Kubota R., Rao R. R., Hwang J., Giordano L., Morinaga A., Okanishi T., Muroyama H., Matsui T., Shao-Horn Y., Eguchi K. (2021). Direct Observation of Surface-Bound
Intermediates during Methanol Oxidation on Platinum under Alkaline
Conditions. J. Phys. Chem. C.

[36] Katayama Y., Nattino F., Giordano L., Hwang J., Rao R. R., Andreussi O., Marzari N., Shao-Horn Y. (2019). An in Situ
Surface-Enhanced Infrared Absorption Spectroscopy Study of Electrochemical
CO2 Reduction: Selectivity Dependence on Surface C-Bound and O-Bound
Reaction Intermediates. J. Phys. Chem. C.

[37] Lim H. K., Lee H., Kim H. (2016). A Seamless
Grid-Based Interface for Mean-Field QM/MM
Coupled with Efficient Solvation Free Energy Calculations. J. Chem. Theory Comput..

[38] Giannozzi P., Baroni S., Bonini N., Calandra M., Car R., Cavazzoni C., Ceresoli D., Chiarotti G. L., Cococcioni M., Dabo I., Dal Corso A., De Gironcoli S., Fabris S., Fratesi G., Gebauer R., Gerstmann U., Gougoussis C., Kokalj A., Lazzeri M., Martin-Samos L., Marzari N., Mauri F., Mazzarello R., Paolini S., Pasquarello A., Paulatto L., Sbraccia C., Scandolo S., Sclauzero G., Seitsonen A. P., Smogunov A., Umari P., Wentzcovitch R. M. (2009). QUANTUM
ESPRESSO: A Modular and Open-Source Software Project for Quantumsimulations
of Materials. J. Phys.: Condens. Matter.

[39] Plimpton S. (1995). Fast Parallel
Algorithms for Short-Range Molecular Dynamics. J. Comput. Phys..

[40] Li G., Zhang B., Rao J., Herranz Gonzalez D., Blake G. R., de Groot R. A., Palstra T. T. M. (2015). Electronic Supporting
Information (ESI): The Effect of Vacancies on Magnetism, Electrical
Transport and Thermoelectric Performance of Marcasite FeSe 2-δ
(Δ=0.05). Chem. Mater..

[41] Perdew J. P., Burke K., Ernzerhof M. (1996). Generalized
Gradient Approximation
Made Simple. Phys. Rev. Lett..

[42] Price D. J., Brooks C. L. (2004). A Modified TIP3P Water Potential
for Simulation with
Ewald Summation. J. Chem. Phys..

[43] Fyta M., Netz R. R. (2012). Ionic Force Field
Optimization Based on Single-Ion
and Ion-Pair Solvation Properties: Going beyond Standard Mixing Rules. J. Chem. Phys..

[44] Bonthuis D.
J., Mamatkulov S. I., Netz R. R. (2016). Optimization of Classical Nonpolarizable
Force Fields for OH- and H3O+. J. Chem. Phys..

[45] Rappe A. K., Casewit C.J., Colwell K. S., Goddard W. A., Skiff W. M. (1992). UFF, a
Full Periodic Table Force Field for Molecular Mechanics and Molecular
Dynamics Simulations. J. Am. Chem. Soc..

[46] Joung I. S., Cheatham T. E. (2008). Determination of Alkali and Halide
Monovalent Ion Parameters
for Use in Explicitly Solvated Biomolecular Simulations. J. Phys. Chem. B.

[47] Hoover W. G. (1985). Canonical
Dynamics: Equilibrium Phase-Space Distributions. Phys. Rev. A.

[48] Nosé S. (1984). A Unified
Formulation of the Constant Temperature Molecular Dynamics Methods. J. Chem. Phys..

[49] Yeh I. C., Berkowitz M. L. (1999). Ewald Summation for Systems with Slab Geometry. J. Chem. Phys..

[50] Nørskov J. K., Rossmeisl J., Logadottir A., Lindqvist L., Kitchin J. R., Bligaard T., Jónsson H. (2004). Origin of
the Overpotential for Oxygen Reduction at a Fuel-Cell Cathode. J. Phys. Chem. B.

[51] Li H., Jensen J. H. (2002). Partial Hessian Vibrational Analysis: The Localization
of the Molecular Vibrational Energy and Entropy. Theor. Chem. Acc..

[52] Lin S. T., Maiti P. K., Goddard W. A. (2010). Two-Phase Thermodynamic
Model for
Efficient and Accurate Absolute Entropy of Water from Molecular Dynamics
Simulations. J. Phys. Chem. B.

[53] Pascal T. A., Lin S.-T., Goddard W. A. (2010). Thermodynamics
of Liquids: Standard
Molar Entropies and Heat Capacities of Common Solvents from 2PT Molecular
Dynamics. Phys. Chem. Chem. Phys..

[54] Kuo D. Y., Kawasaki J. K., Nelson J. N., Kloppenburg J., Hautier G., Shen K. M., Schlom D. G., Suntivich J. (2017). Influence
of Surface Adsorption on the Oxygen Evolution Reaction on IrO2(110). J. Am. Chem. Soc..

[55] Niu J., Conway B. E., Pell W. G. (2004). Comparative Studies of Self-Discharge
by Potential Decay and Float-Current Measurements at C Double-Layer
Capacitor and Battery Electrodes. J. Power Sources.

[56] Sarabia F. J., Sebastián-Pascual P., Koper M. T. M., Climent V., Feliu J. M. (2019). Effect of the Interfacial
Water Structure on the Hydrogen
Evolution Reaction on Pt(111) Modified with Different Nickel Hydroxide
Coverages in Alkaline Media. ACS Appl. Mater.
Interfaces.

[57] Huang H., Chang Y.-C., Huang Y.-C., Li L., Komarek A. C., Tjeng L. H., Orikasa Y., Pao C.-W., Chan T.-S., Chen J.-M., Haw S.-C., Zhou J., Wang Y., Lin H.-J., Chen C.-T., Dong C.-L., Kuo C.-Y., Wang J.-Q., Hu Z., Zhang L. (2023). Unusual Double
Ligand
Holes as Catalytic Active Sites in LiNiO 2. Nat. Commun..

[58] Choe C., Lademann J., Darvin M. E. (2016). Depth Profiles
of Hydrogen Bound
Water Molecule Types and Their Relation to Lipid and Protein Interaction
in the Human Stratum Corneum in Vivo. Analyst.

[59] Yang Z., Li Q., Chou K. C. (2009). Structures
of Water Molecules at the Interfaces of
Aqueous Salt Solutions and Silica: Cation Effects. J. Phys. Chem. C.

[60] Li C. Y., Le J. B., Wang Y. H., Chen S., Yang Z. L., Li J. F., Cheng J., Tian Z. Q. (2019). In Situ Probing
Electrified Interfacial Water Structures at Atomically Flat Surfaces. Nat. Mater..

[61] Du Q., Freysz E., Shen Y. R. (1994). Vibrational Spectra of Water Molecules
at Quartz/Water Interfaces. Phys. Rev. Lett..

[62] Velasco-Velez J. J., Wu C. H., Pascal T. A., Wan L. F., Guo J., Prendergast D., Salmeron M. (2014). The Structure of Interfacial Water
on Gold Electrodes Studied by X-Ray Absorption Spectroscopy. Science.

[63] Li P., Jiang Y., Hu Y., Men Y., Liu Y., Cai W., Chen S. (2022). Hydrogen Bond Network Connectivity in the Electric
Double Layer Dominates the Kinetic PH Effect in Hydrogen Electrocatalysis
on Pt. Nat. Catal..

[64] Dunwell M., Yan Y., Xu B. A. (2016). Surface-Enhanced
Infrared Absorption Spectroscopic
Study of PH Dependent Water Adsorption on Au. Surf. Sci..

[65] Ataka K. I., Yotsuyanagi T., Osawa M. (1996). Potential-Dependent Reorientation
of Water Molecules at an Electrode/Electrolyte Interface Studied by
Surface-Enhanced Infrared Absorption Spectroscopy. J. Phys. Chem..

[66] Dai F., Zhuang Q., Huang G., Deng H., Zhang X. (2023). Infrared Spectrum
Characteristics and Quantification of OH Groups in Coal. ACS Omega.

[67] Gauthier J. A., Dickens C. F., Chen L. D., Doyle A. D., Nørskov J. K. (2017). Solvation
Effects for Oxygen Evolution Reaction Catalysis on IrO 2 (110). J. Phys. Chem. C.

[68] Ledezma-Yanez I., Wallace W. D. Z., Sebastián-Pascual P., Climent V., Feliu J. M., Koper M. T. M. (2017). Interfacial Water
Reorganization
as a PH-Dependent Descriptor of the Hydrogen Evolution Rate on Platinum
Electrodes. Nat. Energy.

[69] Ganassin A., Sebastian P., Climent V., Schuhmann W., Bandarenka A. S., Feliu J. (2017). On the PH Dependence of the Potential
of Maximum Entropy of Ir(111) Electrodes. Sci.
Rep..

[70] Sarpey T. K., Himmelreich A. V., Song K. T., Gubanova E. L., Bandarenka A. S. (2024). The Electrocatalytic
Activity of Au Electrodes Changes Significantly in Various Na+/K+
Supporting Electrolyte Mixtures. Small Sci..

