# Supplementary material for: Multimodal Operando Characterization of Cation Effects at the Iridium Oxide–Electrolyte Interface for Alkaline Water Oxidation
Source: ACS Appl Mater Interfaces. 2026 Mar 4;18(10):15699–710. doi: 10.1021/acsami.5c22249 (PMC13006944; doi:10.1021/acsami.5c22249)
Supplement: Supplementary file 1 [file am5c22249_si_001.pdf]

SUPPORTING INFORMATION

Multimodal *Operando* Characterization of Cation Effects at the Iridium Oxide–Electrolyte Interface for Alkaline Water Oxidation

Yemin Tao,[1] Tomohiko Utsunomiya,[2] Haiting Yu,[3] Seung-Jae Shin,[4] Caiwu Liang,[1,5] Yifeng Wang,[1] Aron Walsh,[1] James R. Durrant,[5] Mary P. Ryan,[1] Yu Katayama,[2] Aliaksandr S. Bandarenka,*[3] Reshma R. Rao.*[1,6]

[1] Department of Materials, Imperial College London, Exhibition Road, SW7 2AZ, London, United Kingdom
[2] Department of Energy and Environmental Materials, SANKEN, The University of Osaka, Mihogaoka 8-1, Osaka 567-0047, Ibaraki, Japan
[3] Department of Physics, Technical University of Munich, James-Franck-Straße 1, 85748 Garching b. München, Germany
[4]School of Energy and Chemical Engineering, Ulsan National Institute of Science and Technology (UNIST), Ulsan 44919, Republic of Korea
[5] Department of Chemistry, Imperial College London, White City, W12 0BZ, London, United Kingdom
[6] Grantham Institute – Climate Change and the Environment, Imperial College London, Exhibition Road, SW7 2AZ, London, United Kingdom

**e-mail: bandarenka@ph.tum.de, reshma.rao@imperial.ac.uk*

1. Characterization of the electrodeposited IrO_x film

Figures S1a and S1b show SEM images of the as-deposited IrO_x film. The film exhibits a rough, nanogranular surface morphology without discernible crystalline facets, consistent with the amorphous nature of IrO_x reported in our previous studies.[1,2] For comparison, the morphology of the bare FTO substrate is shown in Figure S1c. Comparison of Figures S1b and S1c confirms that the IrO_x film fully and uniformly covers the substrate. Figure S2 presents the X-ray diffraction (XRD) pattern of the as-deposited IrO_x film alongside the characteristic diffraction peaks of the FTO substrate. No additional diffraction peaks attributable to crystalline IrO_2 are observed, indicating that the film is XRD-amorphous, in agreement with previous reports from our group.[1]

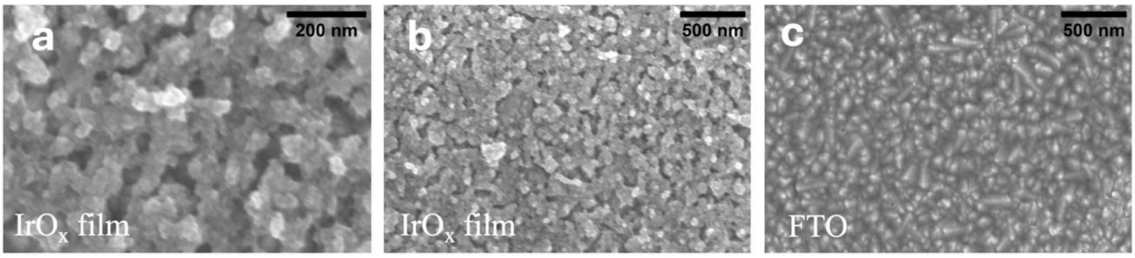

Figure S1: SEM images of (a,b) The as-deposited IrO_x film on FTO substrate with different magnification; (c) FTO substrate.

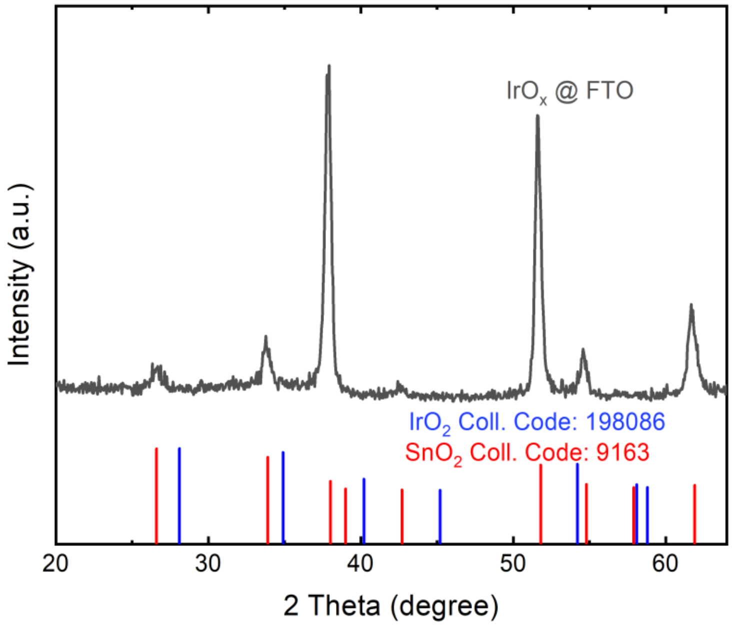

Figure S2: XRD patterns of as-deposited IrO_x on FTO substrate.

Electrochemical activity measurements were conducted both with and without an inert atmosphere. The results are shown in Figure S3. As illustrated in Figure S3a, cyclic voltammograms of electrodeposited IrO_x films on FTO recorded at 10 mV s^{-1} in 0.1 M KOH with and without N_2 purging nearly overlap, indicating negligible differences. This similarity between purged and unpurged conditions is consistently observed across all electrolytes studied (Figure S3b).

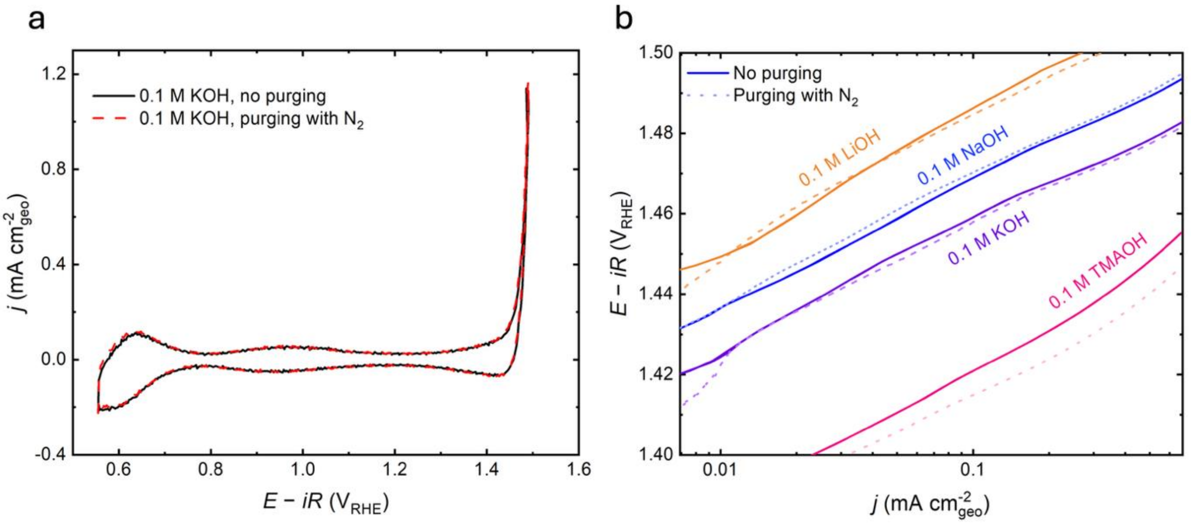

Figure S3. (a) Cyclic voltammograms of electrodeposited IrO_x films on FTO substrates recorded at 10 mV/s in 0.1 M KOH, comparing measurements with N_2 purging (red dotted line) and without purging (black solid line). A Pt coil and Hg/HgO (1 M KOH) were used as the counter and reference electrodes, respectively. (b) Capacitance-corrected cyclic voltammograms in 0.1 M TMAOH (pink), 0.1 M KOH (purple), 0.1 M NaOH (blue), 0.1 M LiOH (orange). Dotted lines indicate measurements under inert conditions with continuous N_2 purging, while solid lines correspond to measurements without purging.

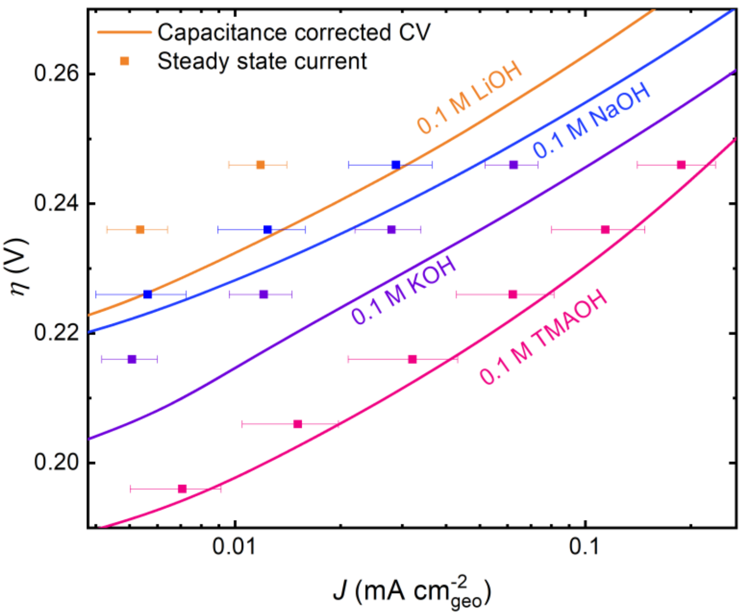

Figure S4. Overpotential as a function of current density for in 0.1 M TMAOH (pink), 0.1 M KOH (purple), 0.1 M NaOH (blue), 0.1 M LiOH (orange).

2. Potential-dependent energetics and kinetics

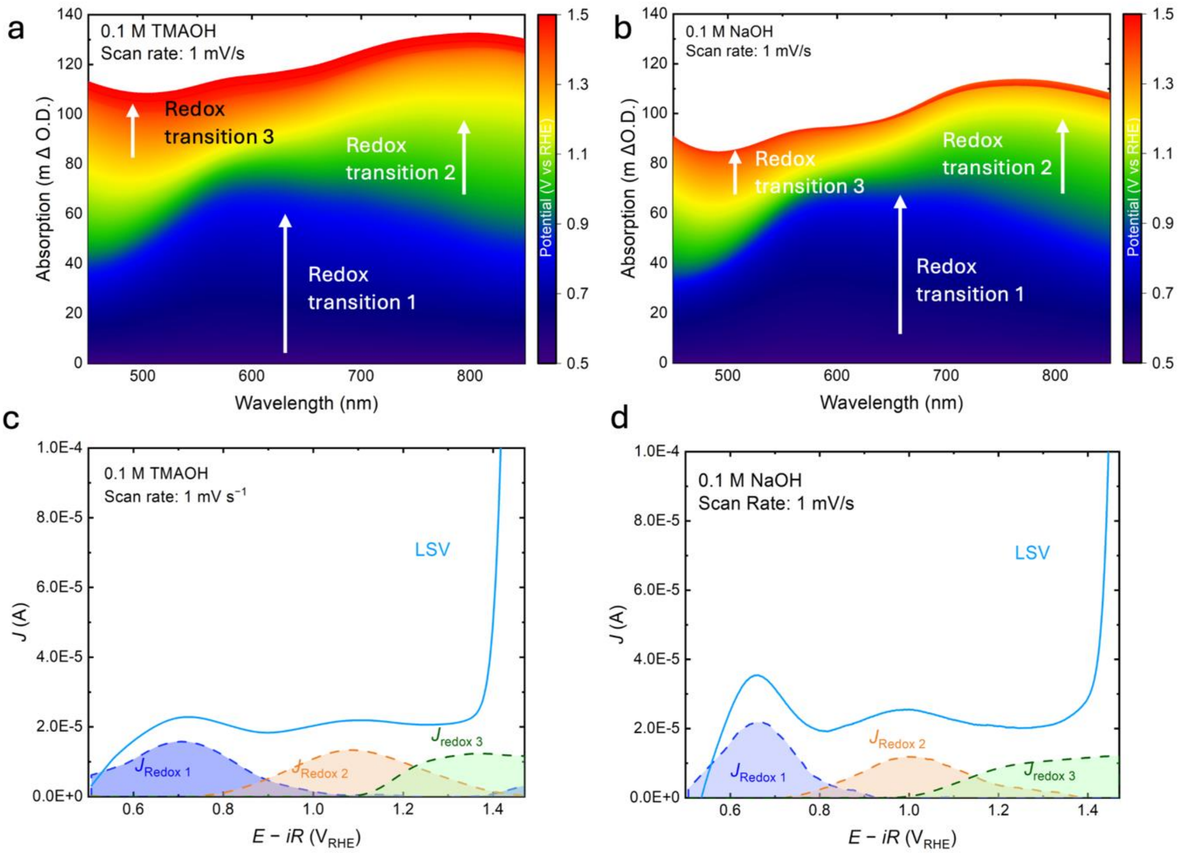

Figure S5: (a, b) differential optical absorption spectra for 0.1 M (a) TMAOH, (d) NaOH during a linear sweep from 0.5 V_{RHE} to 1.5 V_{RHE}, at a scan rate of 1 mV/s. Three distinctive absorption features could be identified at 650nm, 800 nm, and 500 nm with increasing potentials. (c, d) Charging currents (dashed lines) of redox transition 1(blue), 2 (orange), 3 (green) plotted as a function of potential, deconvoluted from optical spectra, in 0.1 M (c) TMAOH, (d) NaOH; the areas under these dotted lines indicate the charges transferred during these processes. The electrochemical current signal is shown in a solid blue line. The charging currents of three redox transitions are comparable to the current from linear sweep voltammetry.

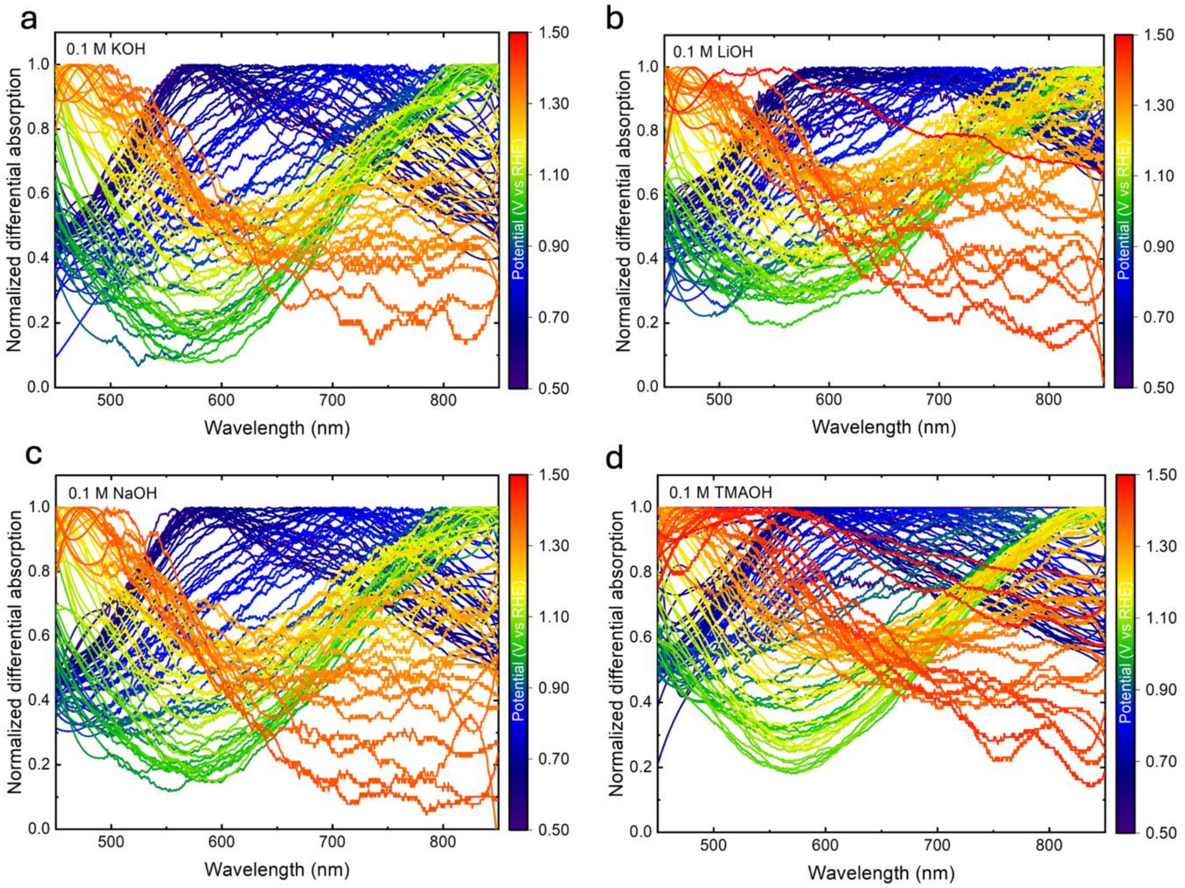

Figure S6: (a) Normalized differential spectra for 0.1 M KOH. This is done by calculating the difference between 2 adjacent optical spectra at a 10-mV interval, then plotting the normalized differentials. The individual spectra corresponding to redox transitions were extracted from potential regions where the spectral shape remained constant. This suggests that changes in absorption were primarily due to variations in the concentration of specific species. The characteristic absorption features for redox transitions 1, 2, and 3 were selected at the difference between 0.65-0.66 V_{RHE}, 0.98-0.99 V_{RHE}, and 1.41-1.42 V_{RHE}, respectively. (b-d) Differential analysis at a 10-mV interval in (b) 0.1 M LiOH, (c) 0.1 M TMAOH, (d) 0.1 M NaOH; where all the characteristic absorption features corresponding to redox transitions 1, 2, and 3 were identified from the same region as above, the differential spectra at potential intervals of 0.65-0.66 V_{RHE}, 0.98-0.99 V_{RHE}, and 1.41-1.42 V_{RHE}, respectively.

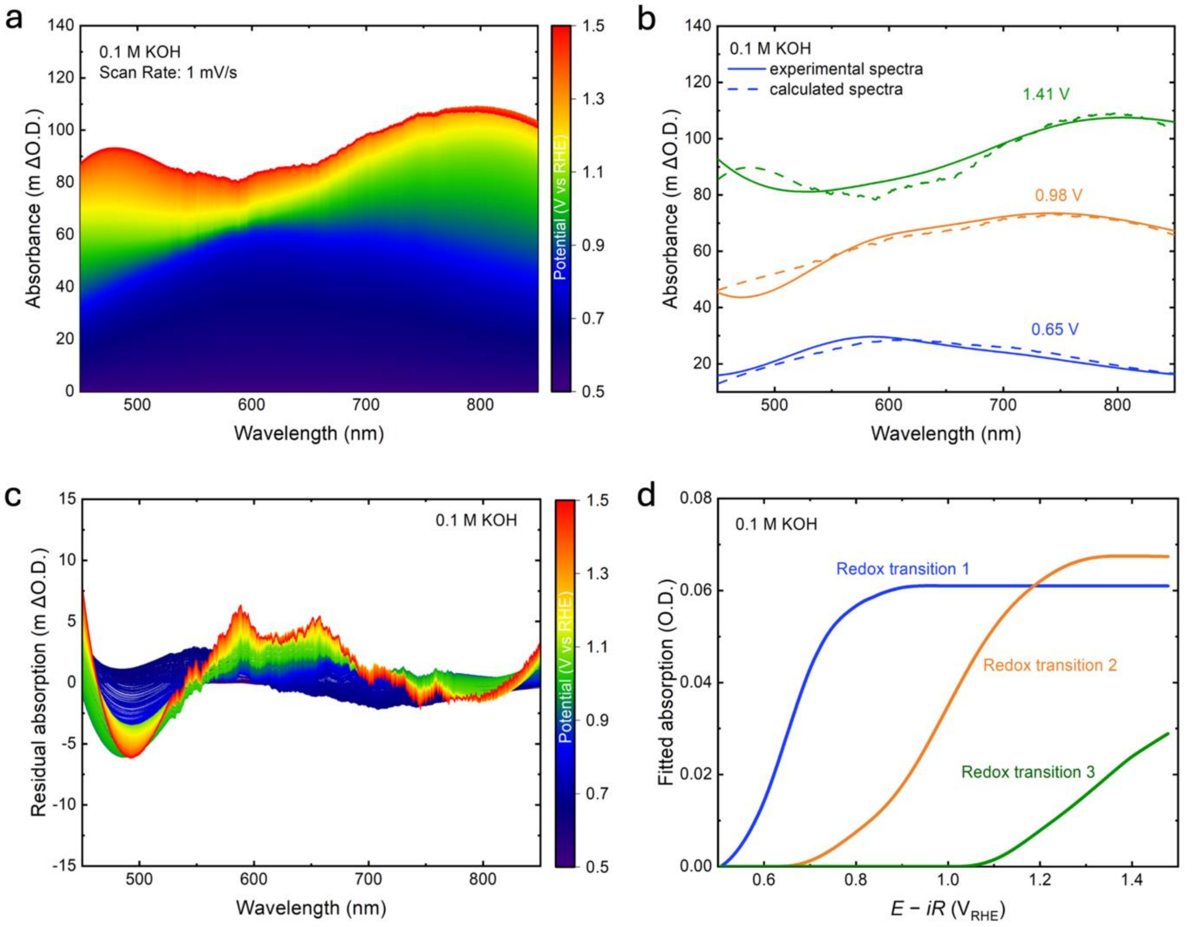

Figure S7: Deconvolution results for IrO_x in 0.1 M KOH. (a) Calculated differential optical spectra at 1 mV intervals over a potential region of 0.5 V_{RHE} to 1.5 V_{RHE}. (b) Comparison between experimental spectra and calculated spectra, sharing very similar features. (c) Fitting residuals generated from the difference between calculated and experimental spectra at every potential. (d) Calculated absorption at its peak wavelength of each redox transition against potential.

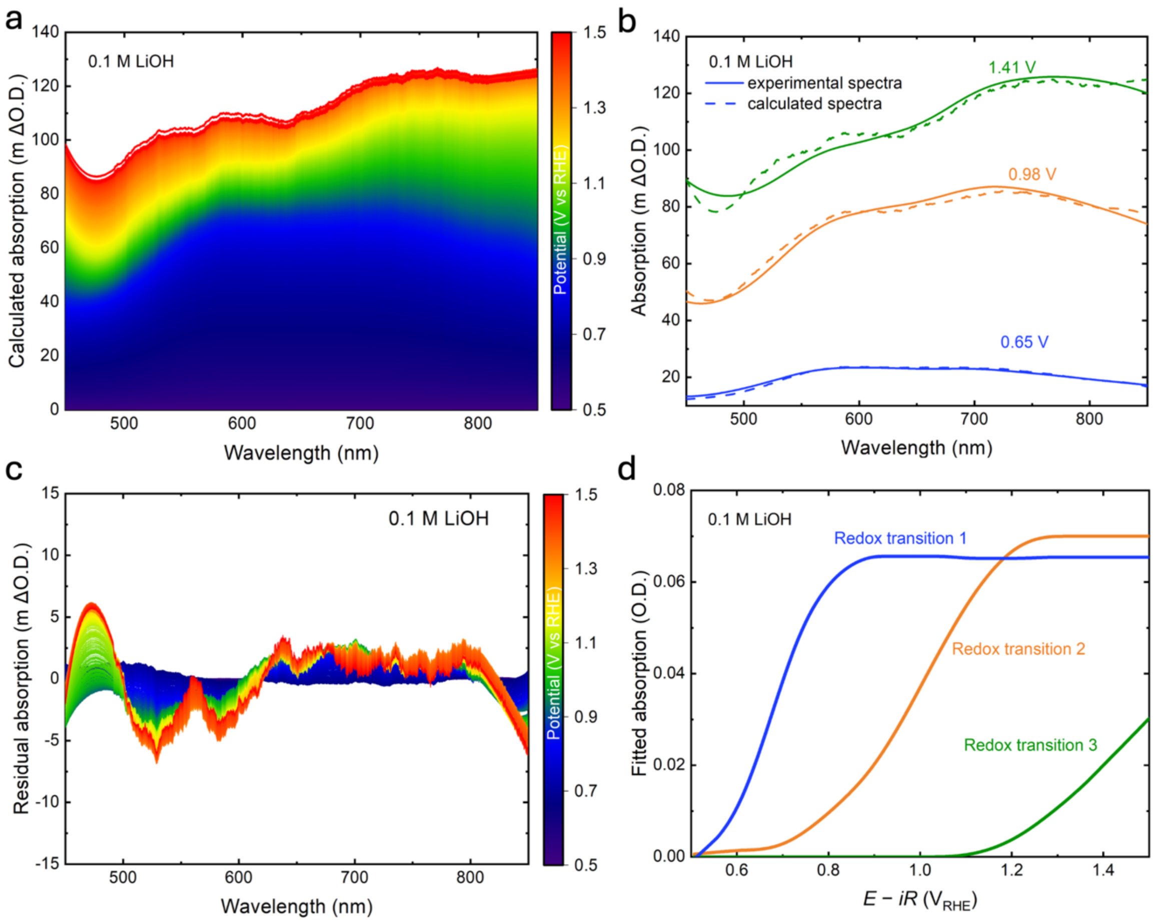

Figure S8: Deconvolution results for IrO_x in 0.1 M LiOH. (a) calculated differential optical spectra at 1 mV intervals over a potential region of 0.5 V_{RHE} to 1.5 V_{RHE}. (b) Comparison between experimental spectra and calculated spectra, sharing very similar features. (c) Fitting residuals generated from the difference between calculated and experimental spectra at every potential. (d) Calculated absorption at its peak wavelength of each redox transition against potential.

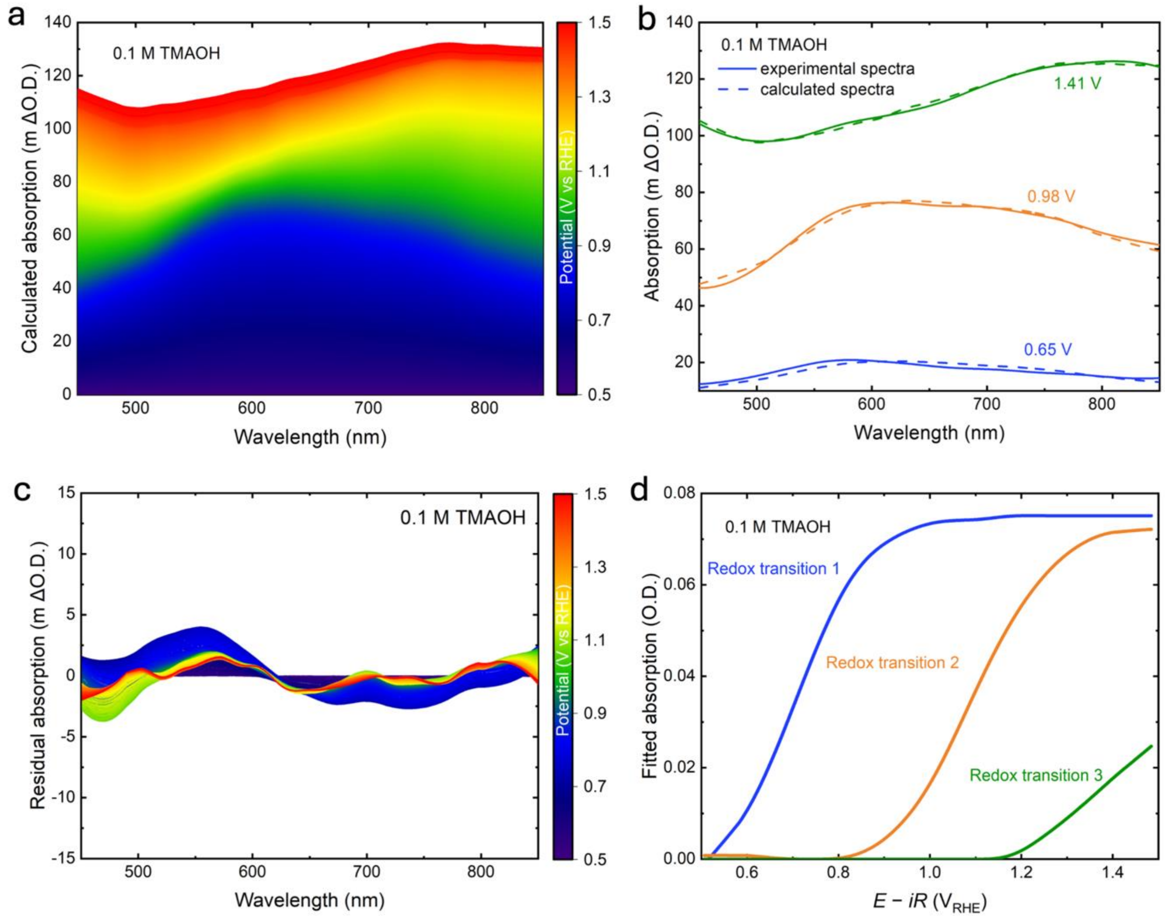

Figure S9: Deconvolution results for IrO_x in 0.1 M TMAOH. (a) calculated differential optical spectra at 1 mV intervals over a potential region of 0.5 V_{RHE} to 1.5 V_{RHE}. (b) Comparison between experimental spectra and calculated spectra, sharing very similar features. (c) Fitting residuals generated from the difference between calculated and experimental spectra at every potential. (d) Calculated absorption at its peak wavelength of each redox transition against potential.

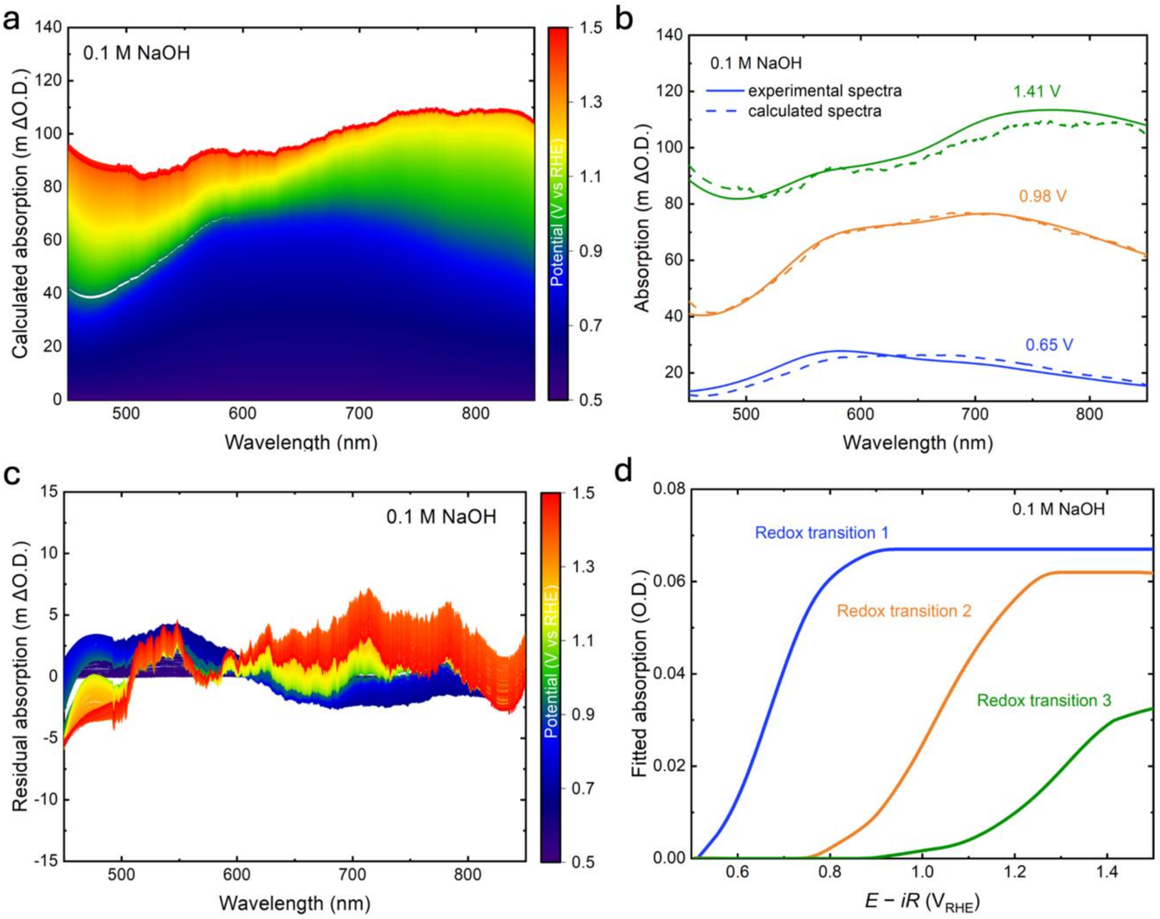

Figure S10: Deconvolution results for IrO_x in 0.1 M NaOH. (a) calculated differential optical spectra at 1 mV intervals over a potential region of 0.5 V_{RHE} to 1.5 V_{RHE}. (b) Comparison between experimental spectra and calculated spectra, sharing very similar features. (c) Fitting residuals generated from the difference between calculated and experimental spectra at every potential. (d) Calculated absorption at its peak wavelength of each redox transition against potential.

3. Extinction coeffcient calculation

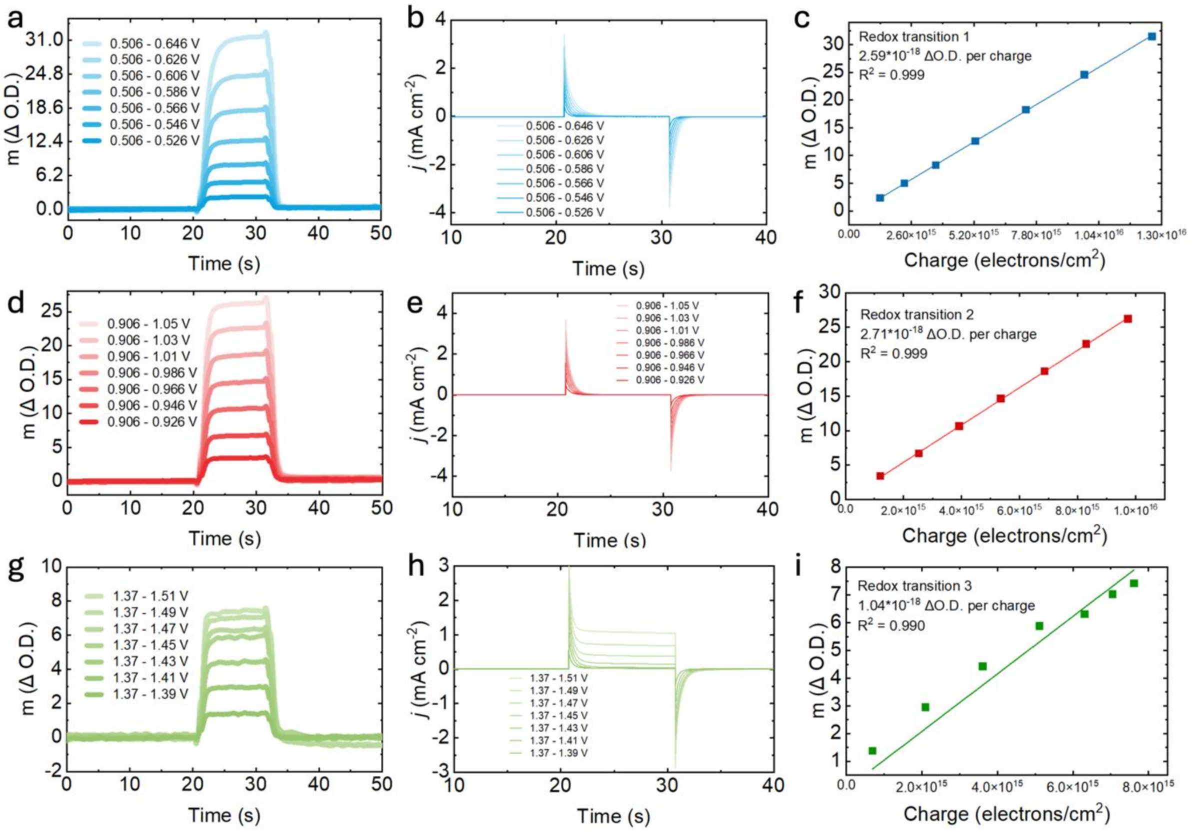

Figure S11: Measuring the extinction coefficient using square-wave measurements in 0.1 M KOH. (a) optical absorption changes at 650 nm when applying the stepped potentials. The potential was held at 0.506 V for 20 s, then went to more oxidizing potentials (as indicated) for 10 s, before going back to 0.506 V for another 20 s. (b) The corresponding current response recorded by the potentiostat. The number of charges can also be calculated by integrating the reduction peak. (c) The relationship between optical absorption and extracted charge for redox transition 1. (d-f) The optical absorption, current response, and the relationship between the optical signal and the extracted charge for redox transition 2. (g-i) The optical absorption, current response, and the relationship between the optical signal and the extracted charge for redox transition 3.

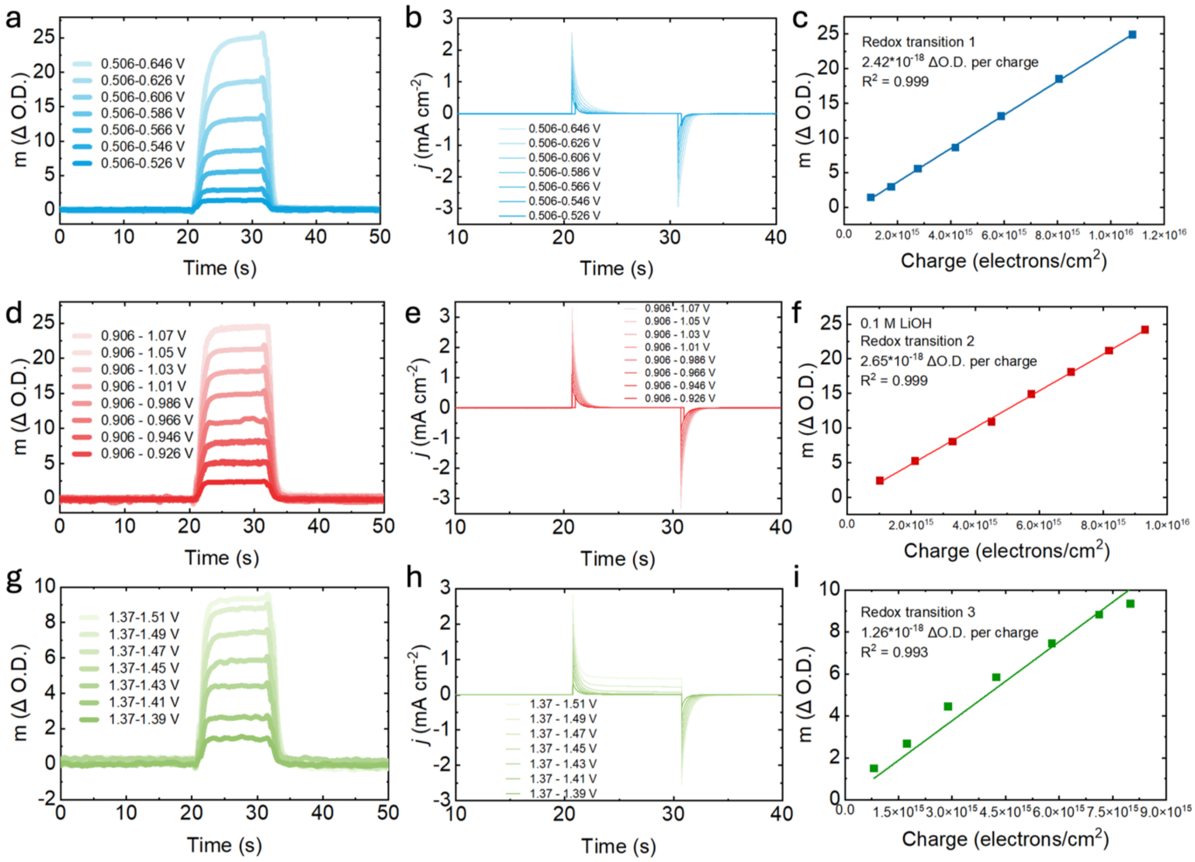

Figure S12: Measuring the extinction coefficient using square-wave measurements in 0.1 M LiOH. (a) optical absorption changes at 650 nm when applying the stepped potentials. The potential was held at 0.506 V for 20 s, then went to more oxidizing potentials (as indicated) for 10 s, before going back to 0.506 V for another 20 s. (b) The corresponding current response recorded by the potentiostat. The number of charges can also be calculated by integrating the reduction peak. (c) The relationship between optical absorption and extracted charge for redox transition 1. (d-f) The optical absorption, current response, and the relationship between the optical signal and the extracted charge for redox transition 2. (g-i) The optical absorption, current response, and the relationship between the optical signal and the extracted charge for redox transition 3.

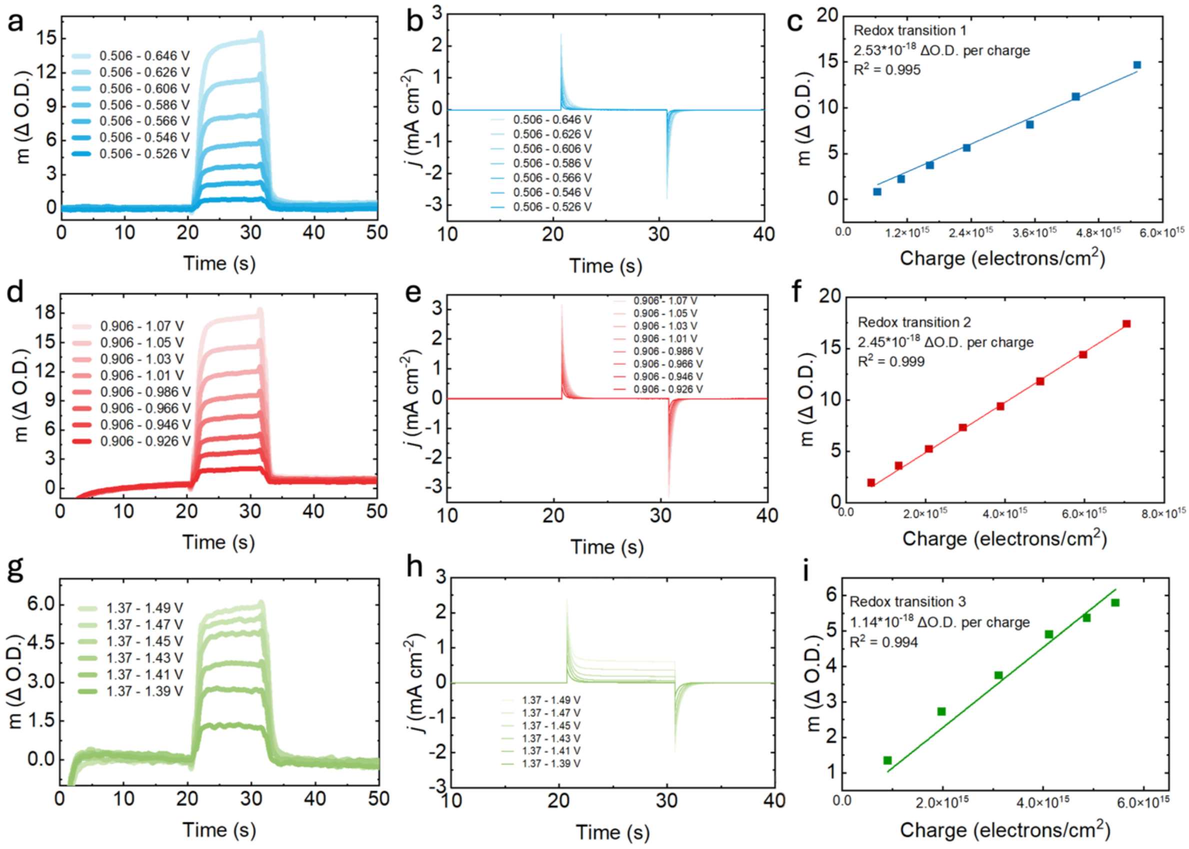

Figure S13: Measuring the extinction coefficient using square-wave measurements in 0.1 M TMAOH. (a) optical absorption changes at 650 nm when applying the stepped potentials. The potential was held at 0.506 V for 20 s, then went to more oxidizing potentials (as indicated) for 10 s, before going back to 0.506 V for another 20 s. (b) The corresponding current response recorded by the potentiostat. The number of charges can also be calculated by integrating the reduction peak. (c) The relationship between optical absorption and extracted charge for redox transition 1. (d-f) The optical absorption, current response and the relationship between the optical signal and the extracted charge for redox transition 2. (g-i) The optical absorption, current response, and the relationship between the optical signal and the extracted charge for redox transition 3.

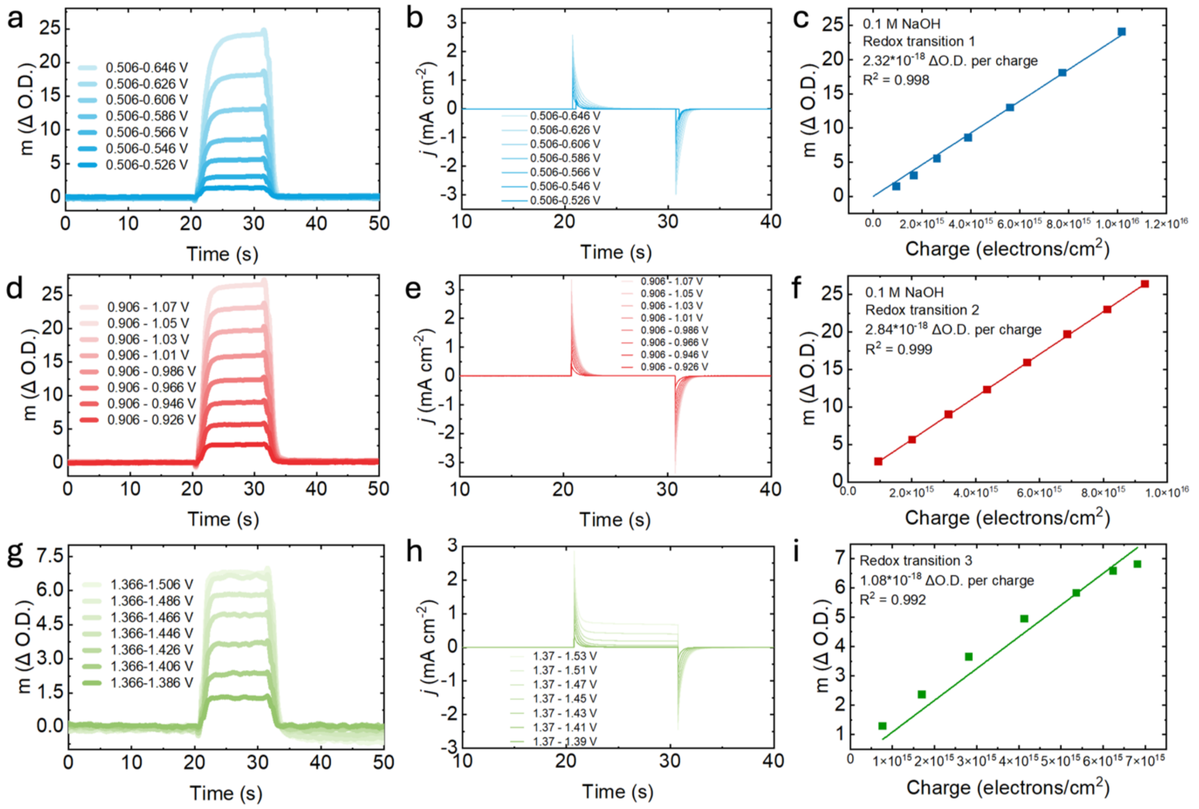

Figure S14: Measuring the extinction coefficient using square-wave measurements in 0.1 M NaOH. (a) optical absorption changes at 650 nm when applying the stepped potentials. The potential was held at 0.506 V for 20 s, then went to more oxidizing potentials (as indicated) for 10 s, before going back to 0.506 V for another 20 s. (b) The corresponding current response recorded by the potentiostat. The number of charges can also be calculated by integrating the reduction peak. (c) The relationship between optical absorption and extracted charge for redox transition 1. (d-f) The optical absorption, current response and the relationship between the optical signal and the extracted charge for redox transition 2. (g-i) The optical absorption, current response, and the relationship between the optical signal and the extracted charge for redox transition 3.

Supplementary note 1: Frumkin isotherm fitting:

The procedure for Frumkin isotherm fitting has been detailed in our previous work[1,3]; a summary is provided here. The free energies of surface adsorbates during redox transitions are given by:

$$\Delta G = \Delta G^0 + RT\ln(\theta/1-\theta)$$

Where θ is the coverage, T is the temperature in Kelvin, and R is the universal gas constant. ΔG^0 is the chemical potential intrinsic to the individual redox transition. In this work, we assume that the maximum concentration observed in optical spectroscopy corresponds to full surface coverage. Accordingly, the coverage is determined by normalizing to this maximum concentration. The interaction parameter is extracted from a Frumkin fit of the electroadsorption isotherms, providing a quantitative measure of the intersite interactions for each redox transition (Figure S15). For this study, we further assume that the maximum concentration of redox transition 3 is identical to that of redox transition 2. Therefore, the coverage of redox transition 3 is calculated by normalizing to the maximum optical density of redox transition 2.

Substituting $\Delta G = -nFU$ in the above equation, we will have

$$U = U^0 - \frac{RT}{nF}\ln(\theta/1-\theta)$$

Where U is the reduction potential and U^0 is the standard reduction potential of the redox transition, F is the Faraday constant, and n is the number of electrons transferred in the reaction. If we write the potentials in both side in reversible hydrogen potential for oxidation reactions, we will get:

$$U_{RHE} = U^0_{RHE} + \frac{RT}{nF}\ln(\theta/1-\theta)$$

Based on previous research in our group, there are non-negligible interactions between absorbates in this system, which makes Langmuir fitting not suitable. Thus, Frumkin isotherm fitting is prioritized.

$$\Delta G^0_\theta = \Delta G^0_{\theta=0} + r\theta$$

Substituting U_{RHE} into the above equation, we have

$$U_{RHE} = U^0_{RHE\,(\theta=0)} + \frac{RT}{nF}\ln(\theta/1-\theta) + \frac{r\theta}{nF}$$

Where r represents the interaction parameter between surface adsorbates, $U^0_{RHE\,(\theta=0)}$ denotes the potential on the reversible hydrogen electrode (RHE) scale at zero coverage, and n is the number of electrons transferred in the reaction, which in this case is one. The fitting is performed within the range $0.05< \theta <0.95$ to ensure greater reliability of the results.

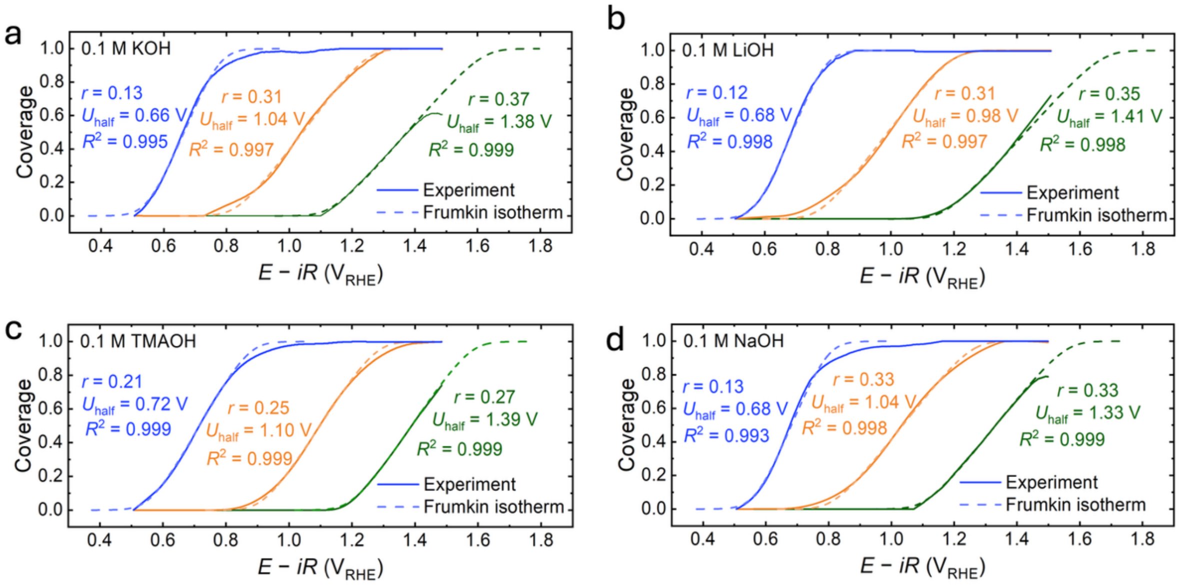

Figure S15: Coverage as a function of potential for (a) 0.1 M KOH, (b) 0.1 M LiOH, (c) 0.1 M TMAOH, (d) 0.1 M NaOH; with redox transition 1 in blue, redox transition 2 in orange and 3 in green. The experimental data are shown in solid lines while the Frumkin isotherm fitting are shown in dashed lines. The coverage is fitted in the region of 0.05-0.95. The fitting yielded R^2 values ranging from 0.993 to 0.999.

4. Potential decay measurements

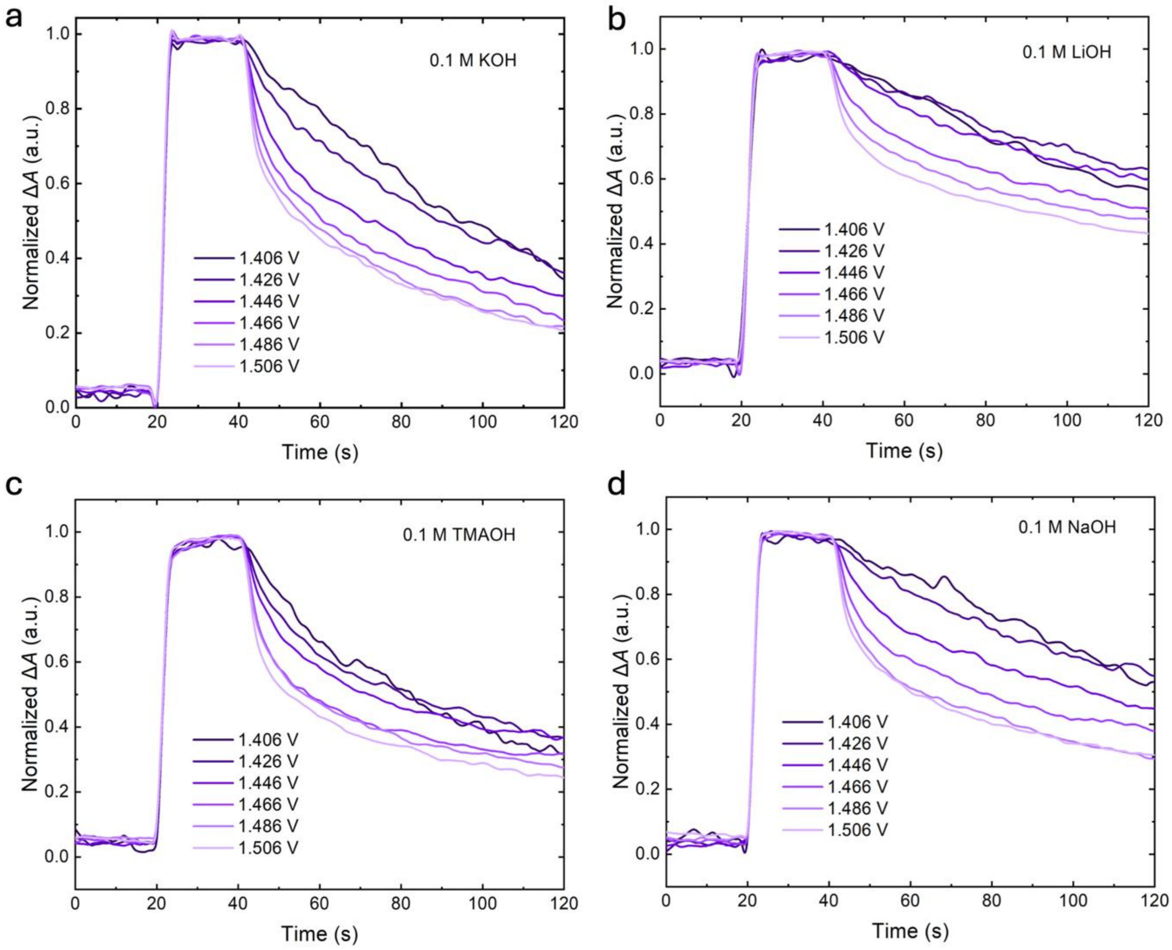

Figure S16: Normalized potential decay plots for (a) 0.1 M KOH, (b) 0.1 M LiOH, (c) 0.1 M TMAOH, (d) 0.1 M NaOH. Data were smoothed using the Lowess method with a window size of around 100 points. Potential decay measurements were done by recording the optical signal when holding the potential at 1.37 V_{RHE} for 20 s, before going to a more oxidizing potential (as indicated on the graphs) for a further 20 s, and then setting the system to open circuit for another 80s.

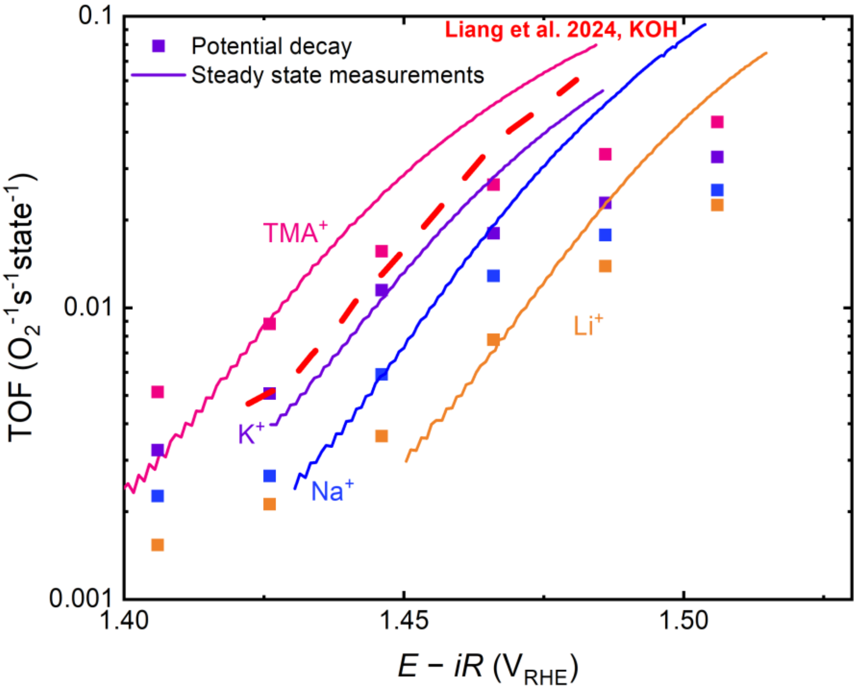

Figure S17: Turnover frequency comparison, where the scattered data represents the results from potential decay measurements and line data shows the results from steady state measurements. The red dashed line shows results from previous literature, where the authors measured turnover frequency in 0.1 M KOH.[3] Our results show good agreement with the previous study.

5. Probing the interfacial water structure

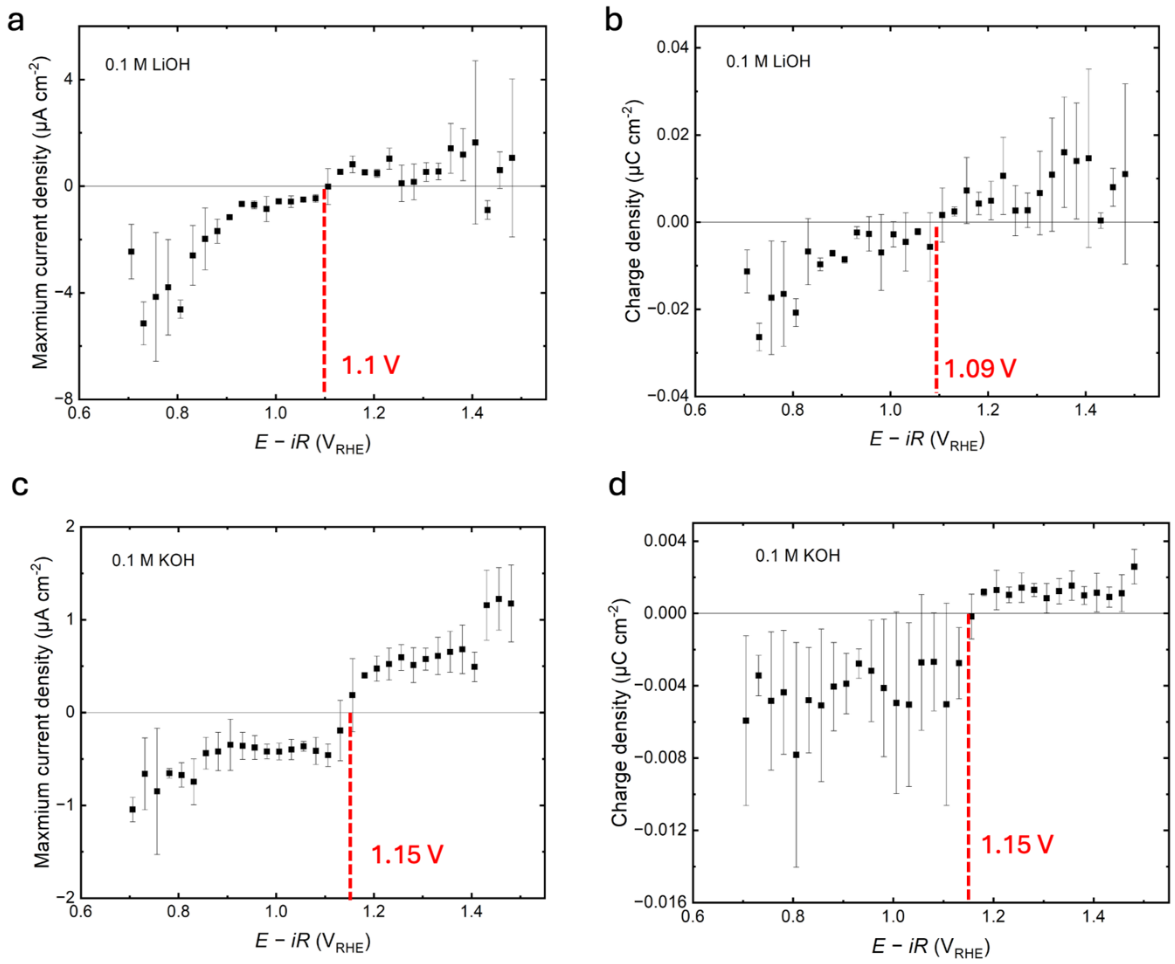

Figure S18: 2D LICT data comparing two approaches for determining the potential of maximum entropy (PME): (i) the point where the transient current density crosses zero and (ii) the point where the charge density is zero. Red lines indicate the PME values, which are consistent across both methods—1.09 V_{RHE} in 0.1 M LiOH and 1.15 V_{RHE} in 0.1 M KOH—demonstrating the reliability of the analysis. The laser was activated following current stabilization and operated in pulsed mode (0.1 s intervals) for 4 s. The transient current data plotted were recorded from 2 s post-irradiation onset to minimize thermal artifacts at each potential.

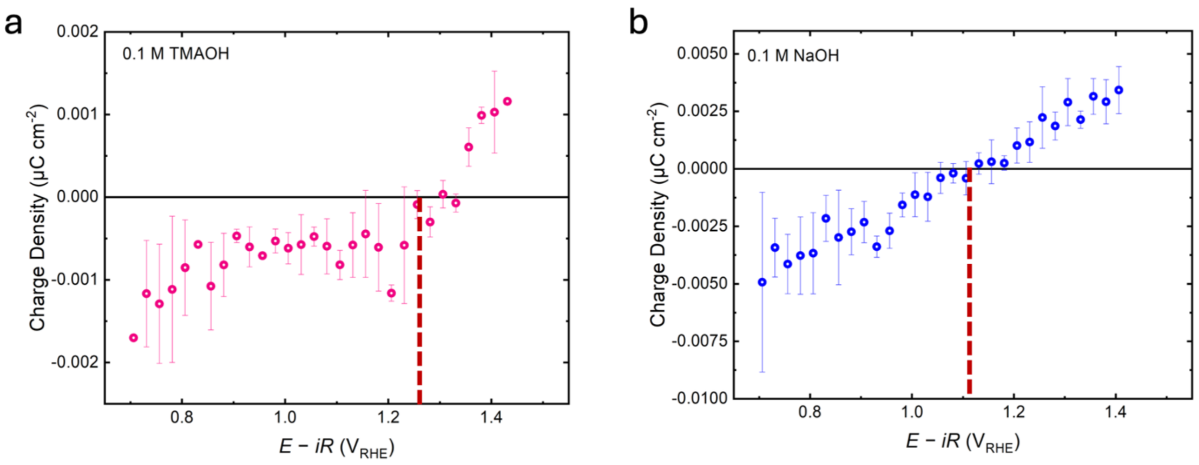

Figure S19: LICT data showing the correlation between charge density and applied potential for (a) 0.1 M TMAOH, (b) 0.1 M NaOH. Potentials of maximum entropy have been indicated using a red line, 1.26 V_{RHE} in 0.1 M TMAOH, 1.12 V_{RHE} in 0.1 M NaOH. The laser was activated following current stabilization and operated in pulsed mode (0.1 s intervals) for 4 s. The transient current data plotted were recorded from 2 s post-irradiation onset to minimize thermal artifacts at each potential.

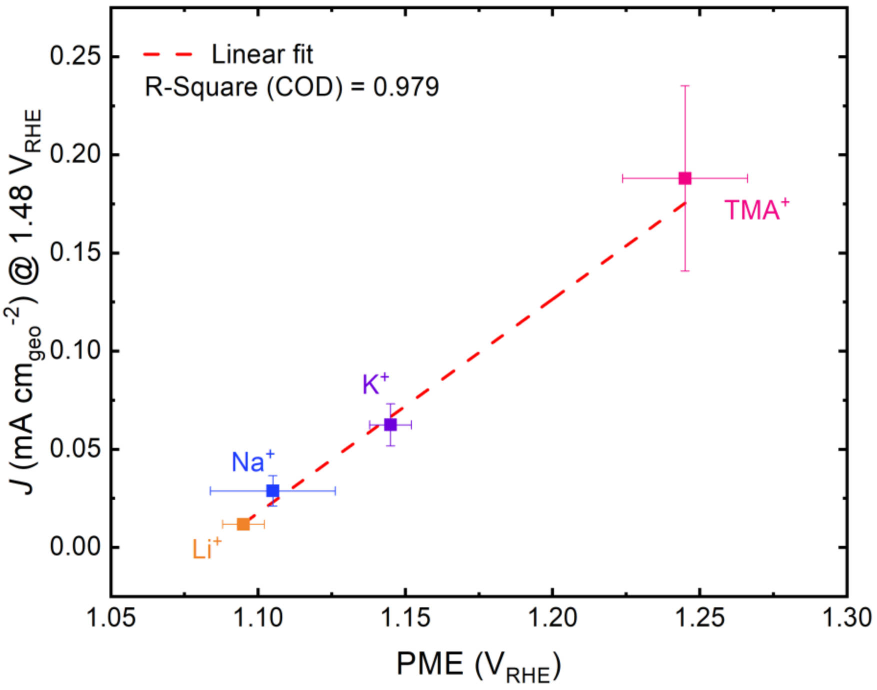

Figure S20: Electrocatalytic activity at 1.48 V_{RHE} as a function of the potential of maximum entropy for IrO_x electrodes in electrolytes containing different cations. The red dashed line represents a linear fit, yielding a high coefficient of determination ($R^2 = 0.979$).

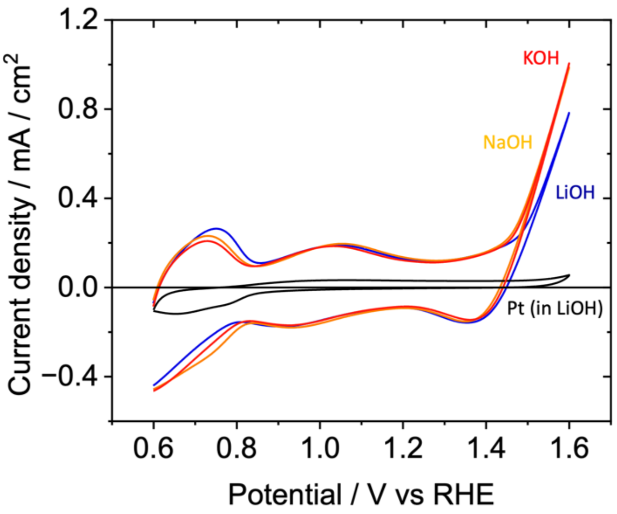

Figure S21: Cyclic voltammograms of electrodeposited IrOx film on Pt coated Si ATR prism, at 10 mV/s in 0.1 M KOH (red), 0.1 M NaOH (orange), 0.1 M LiOH (blue), and Pt in 0.1 M LiOH (black) under room temperature.

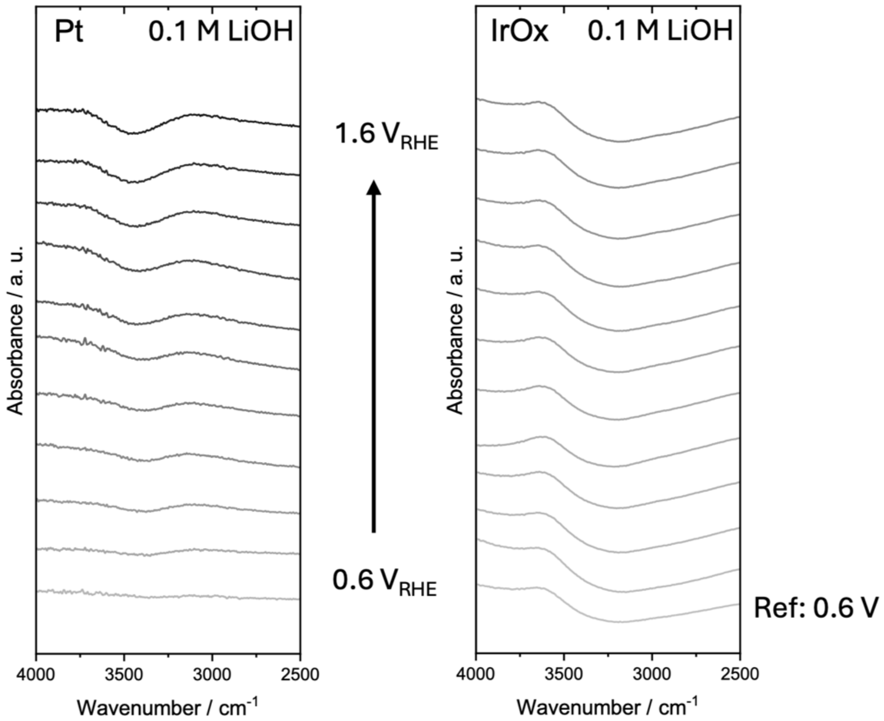

Figure S22: ATR-SEIRAS spectra in the O−H stretching regime, under different applied potentials with the spectra at 0.6 V_{RHE} as reference, in 0.1 M LiOH on bare Pt and IrO_x film. The baseline of each spectrum was corrected using OMNIC software with a three-point autocorrection method.

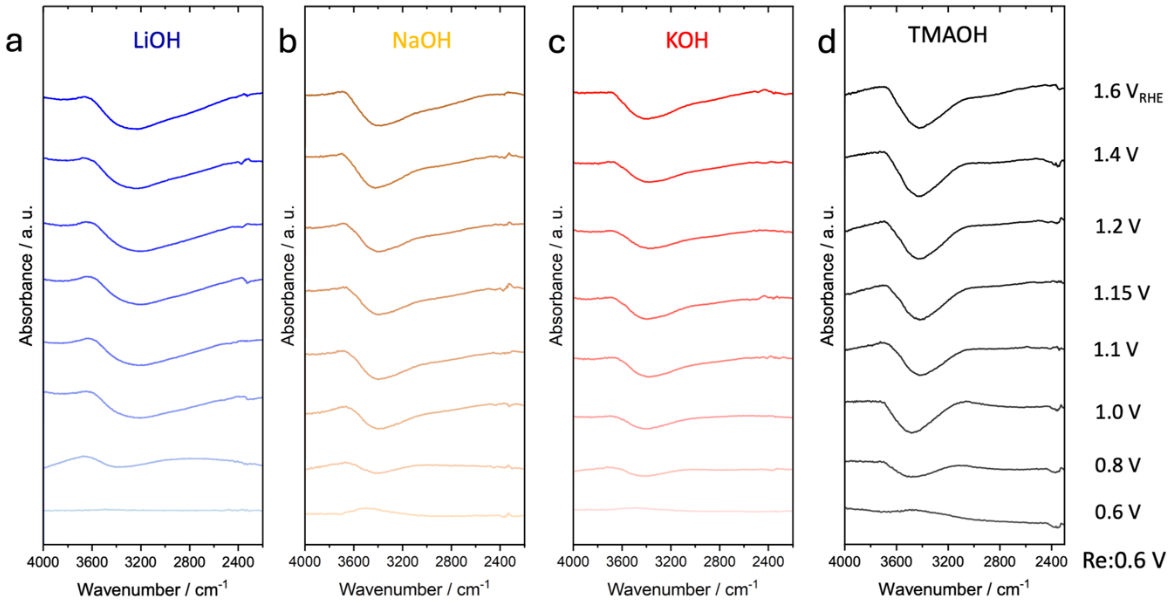

Figure S23: ATR-SEIRAS spectra in the O−H stretching regime, under different applied potentials with the spectra at 0.6 V_{RHE} as reference, in (a) 0.1 M LiOH, (b) 0.1 M NaOH, (c) 0.1 M KOH, (d) 0.1 M TMAOH. The baseline of each spectrum was corrected using OMNIC software with a three-point autocorrection method.

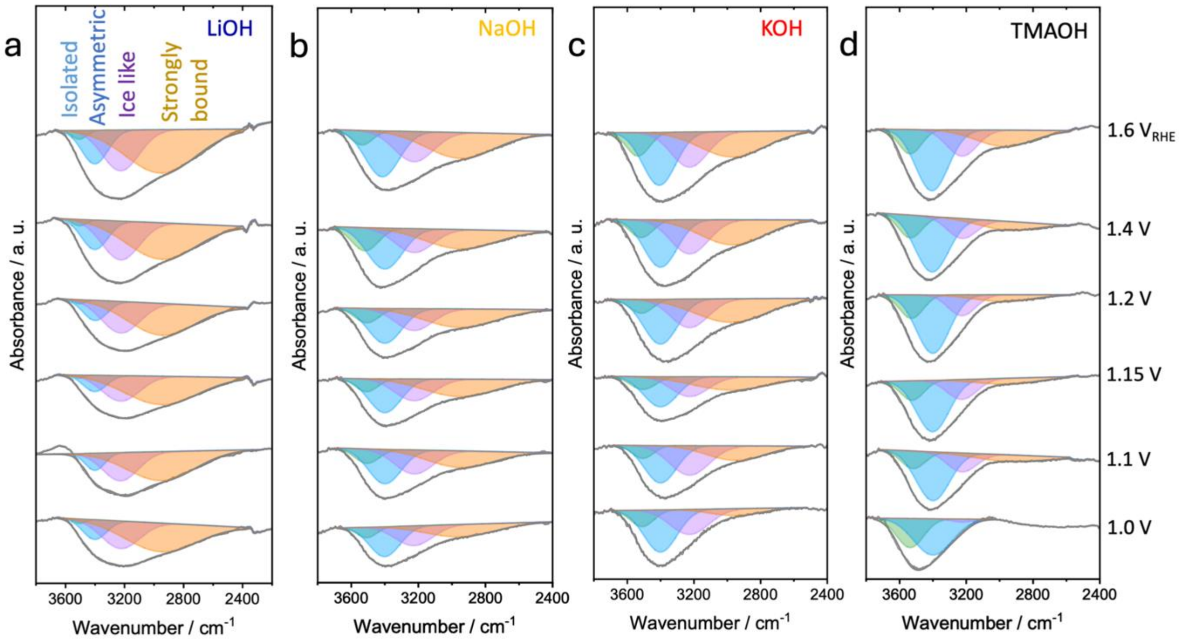

Figure S24: ATR-SEIRAS spectra deconvolution at different potentials, in (a) 0.1 M LiOH, (b) 0.1 M NaOH, (c) 0.1 M KOH, (d) 0.1 M TMAOH.

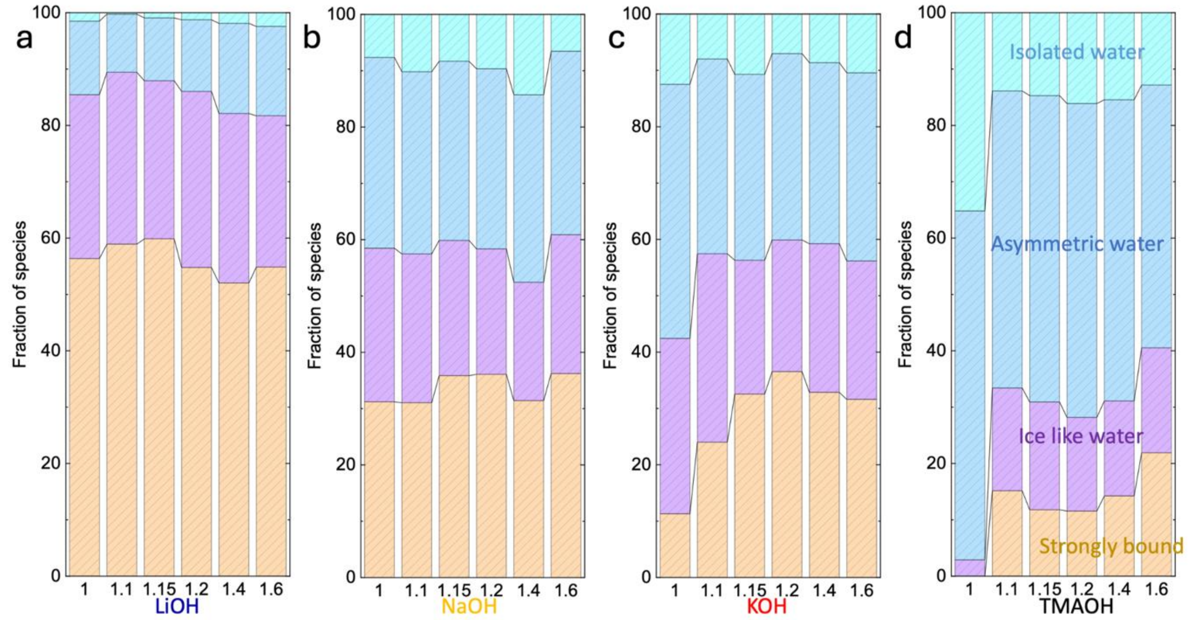

Figure S25: Quantification of interfacial water structures at specified potentials, in (a) 0.1 M LiOH, (b) 0.1 M NaOH, (c) 0.1 M KOH, (d) 0.1 M TMAOH.

Electrolyte	Isolated water		Asymmetric water		Ice like water		Strongly bond water	
0.1 M LiOH	Peak position (μ)	FWHM (σ)	Peak position (μ)	FWHM (σ)	Peak position (μ)	FWHM (σ)	Peak position (μ)	FWHM (σ)
1 V	3506	146.1	3405	214.2	3226	302.2	2969.4	253.3
1.1 V	3506	42.8	3400	157.6	3226	282.7	2950	535.3
1.15 V	3506	79.8	3401.7	186.7	3226	303.3	2950	581.1
1.2 V	3506	89.63	3400	194.2	3226	307	2950	532.6
1.4 V	3506	112.8	3402.5	210.5	3226	301.1	2950	555.5
1.6 V	3506	120	3400.8	209.7	3226	294.3	2950	565.8

Table S1: Summary of Gaussian fitting parameters for the O–H stretching region under different experimental conditions. The leftmost column lists the applied potentials in 0.1 M LiOH electrolyte, while the top row categorizes four types of interfacial water species—isolated, asymmetric, ice-like, and strongly hydrogen-bonded water. For each water species, the corresponding peak position and full width at half maximum (FWHM) obtained from Gaussian fitting are reported.

Electrolyte	Isolated water		Asymmetric water		Ice like water		Strongly bond water	
0.1 M NaOH	Peak position (μ)	FWHM (σ)	Peak position (μ)	FWHM (σ)	Peak position (μ)	FWHM (σ)	Peak position (μ)	FWHM (σ)
1 V	3506	137.9	3400	216.2	3226	262.6	2950	485.4
1.1 V	3506	172.8	3400	232.4	3226	271.8	2950	464.5
1.15 V	3508.2	157.4	3400	231.7	3226	272.5	2950	506
1.2 V	3507.2	172.5	3400.1	239.1	3226	265.2	2950.1	507.5
1.4 V	3518	189.1	3400.4	250.2	3226	269.9	2950	501.9
1.6 V	3536	150.5	3414.5	238.8	3226	274.6	2950.2	473.5

Table S2: Summary of Gaussian fitting parameters for the O–H stretching region under different experimental conditions for 0.1 M NaOH, with peak positions and full width at half maximum (FWHM) for each water species reported.

Electro-lyte	Isolated water		Asymmetric water		Ice like water		Strongly bond water	
0.1 M KOH	Peak position (μ)	FWHM (σ)	Peak position (μ)	FWHM (σ)	Peak position (μ)	FWHM (σ)	Peak position (μ)	FWHM (σ)
1 V	3509.7	166.5	3400	235.6	3226	273.6	2950	360.7
1.1 V	3506	151.4	3400	228.5	3226	279.9	2980	407.7
1.15 V	3506	181.6	3400	241.5	3226	264.3	2980	543
1.2 V	3514.6	163.4	3400	241.2	3226	267.2	2977.5	508.5
1.4 V	3515.9	166.9	3400	235.8	3226	268.8	2968.3	456.9
1.6 V	3535	163.9	3407.6	233.7	3226	258.7	2967.4	460.9

Table S3: Summary of Gaussian fitting parameters for the O–H stretching region under different experimental conditions for 0.1 M KOH, with peak positions and full width at half maximum (FWHM) for each water species reported.

Electrolyte	Isolated water		Asymmetric water		Ice like water		Strongly bond water	
0.1 M TMAOH	Peak position (μ)	FWHM (σ)	Peak position (μ)	FWHM (σ)	Peak position (μ)	FWHM (σ)	Peak position (μ)	FWHM (σ)
1 V	3536	191	3400	269.6	3226	144.5	2980	111.2
1.1 V	3518.2	172.2	3400	235.8	3226	210.2	2950	501.9
1.15 V	3523.9	166.7	3400.4	233.2	3226	212.1	2950	442.8
1.2 V	3528.1	167.6	3400	233.3	3226	194.6	2980	424.6
1.4 V	3536	166.7	3405.3	237.5	3226	206.9	2958.1	399.4
1.6 V	3536	159.7	3405.2	231.6	3225	214.5	2969.6	404.4

Table S4: Summary of Gaussian fitting parameters for the O–H stretching region under different experimental conditions for 0.1 M TMAOH, with peak positions and full width at half maximum (FWHM) for each water species reported.

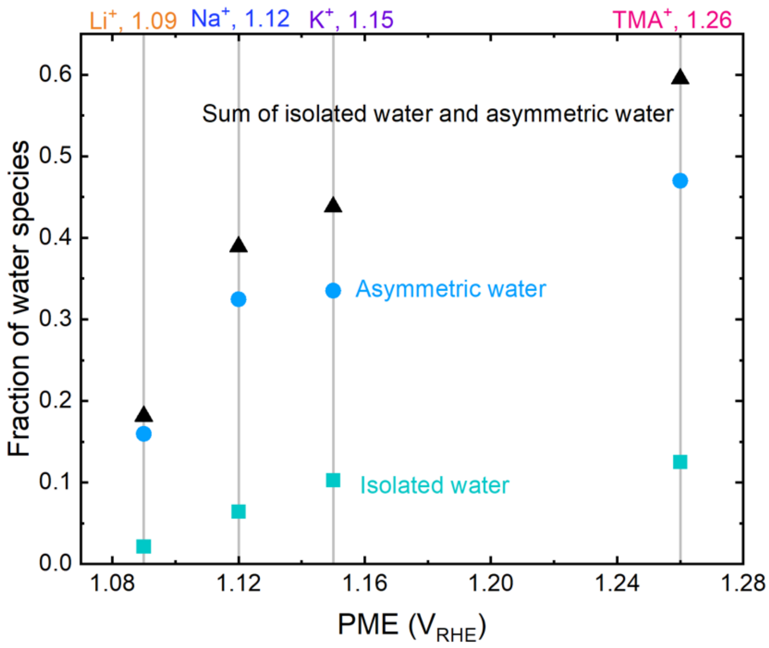

Figure S26: Fraction of species at 1.6 V_{RHE} as a function of PME, where PME is indicated by the grey line with the label above to show the value. It shows the relative abundance of isolated and asymmetric water species increases systematically from Li^+ to Na^+, K^+, and TMA^+, while PME also increases throughout.

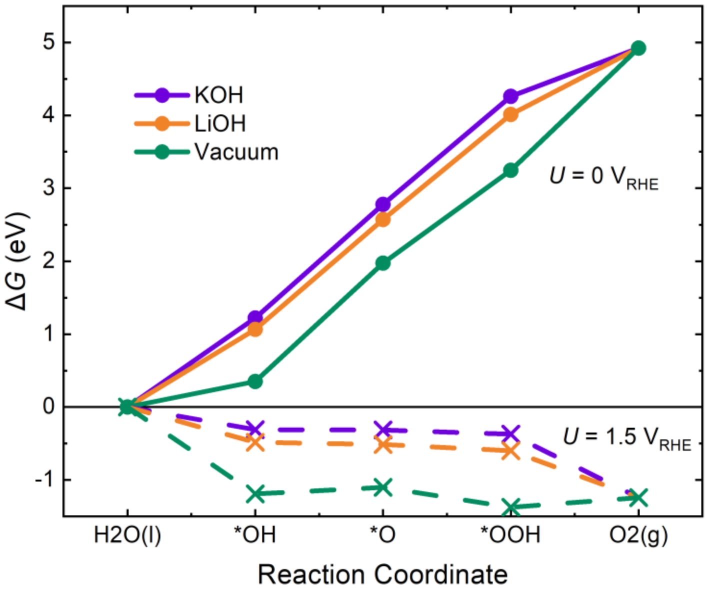

Figure S27: Gibbs free energy difference (ΔG) using different electrolytes or vacuum conditions. The solid lines are at $U = 0.0$ V, and the dashed lines are at $U = 1.5$ V.

Reference:

[1] C. Liang, R. R. Rao, K. L. Svane, J. H. L. Hadden, B. Moss, S. B. Scott, M. Sachs, J. Murawski, A. M. Frandsen, D. J. Riley, M. P. Ryan, J. Rossmeisl, J. R. Durrant, I. E. L. Stephens, “Unravelling the effects of active site density and energetics on the water oxidation activity of iridium oxides” *Nature Catalysis* **2024**, *7*, 763–775.

[2] C. Bozal-Ginesta, R. R. Rao, C. A. Mesa, X. Liu, S. A. J. Hillman, I. E. L. Stephens, J. R. Durrant, “Redox-State Kinetics in Water-Oxidation IrOx Electrocatalysts Measured by Operando Spectroelectrochemistry” *ACS Catal.* **2021**, *11*, 15013–15025.

[3] C. Liang, Y. Katayama, Y. Tao, A. Morinaga, B. Moss, V. Celorrio, M. Ryan, I. E. L. Stephens, J. R. Durrant, R. R. Rao, “Role of Electrolyte pH on Water Oxidation for Iridium Oxides” *J. Am. Chem. Soc.* **2024**, *146*, 8928–8938.
